# Distinct impact of antibiotics on the gut microbiome and resistome: a longitudinal multicenter cohort study

**DOI:** 10.1186/s12915-019-0692-y

**Published:** 2019-09-18

**Authors:** Matthias Willmann, Maria J. G. T. Vehreschild, Lena M. Biehl, Wichard Vogel, Daniela Dörfel, Axel Hamprecht, Harald Seifert, Ingo B. Autenrieth, Silke Peter

**Affiliations:** 1 Institute of Medical Microbiology and Hygiene, University of Tübingen, Tübingen, Germany; 2 German Center for Infection Research (DZIF), partner site Tübingen, Tübingen, Germany; 3 1st Department of Internal Medicine, University Hospital of Cologne, Cologne, Germany; 4 German Center for Infection Research (DZIF), partner site Bonn-Cologne, Cologne, Germany; 5 Medical Center, Department of Hematology, Oncology, Immunology, Rheumatology & Pulmonology, University of Tübingen, Tübingen, Germany; 6 Institute for Medical Microbiology, Immunology and Hygiene, University Hospital of Cologne, Cologne, Germany

**Keywords:** Antimicrobial resistance, Metagenomics study, Resistome analysis, Antibiotic impact prediction, Plasmid expansion

## Abstract

**Background:**

The selection pressure exercised by antibiotic drugs is an important consideration for the wise stewardship of antimicrobial treatment programs. Treatment decisions are currently based on crude assumptions, and there is an urgent need to develop a more quantitative knowledge base that can enable predictions of the impact of individual antibiotics on the human gut microbiome and resistome.

**Results:**

Using shotgun metagenomics, we quantified changes in the gut microbiome in two cohorts of hematological patients receiving prophylactic antibiotics; one cohort was treated with ciprofloxacin in a hospital in Tübingen and the other with cotrimoxazole in a hospital in Cologne. Analyzing this rich longitudinal dataset, we found that gut microbiome diversity was reduced in both treatment cohorts to a similar extent, while effects on the gut resistome differed. We observed a sharp increase in the relative abundance of sulfonamide antibiotic resistance genes (ARGs) by 148.1% per cumulative defined daily dose of cotrimoxazole in the Cologne cohort, but not in the Tübingen cohort treated with ciprofloxacin. Through multivariate modeling, we found that factors such as individual baseline microbiome, resistome, and plasmid diversity; liver/kidney function; and concurrent medication, especially virostatic agents, influence resistome alterations. Strikingly, we observed different effects on the plasmidome in the two treatment groups. There was a substantial increase in the abundance of ARG-carrying plasmids in the cohort treated with cotrimoxazole, but not in the cohort treated with ciprofloxacin, indicating that cotrimoxazole might contribute more efficiently to the spread of resistance.

**Conclusions:**

Our study represents a step forward in developing the capability to predict the effect of individual antimicrobials on the human microbiome and resistome. Our results indicate that to achieve this, integration of the individual baseline microbiome, resistome, and mobilome status as well as additional individual patient factors will be required. Such personalized predictions may in the future increase patient safety and reduce the spread of resistance.

**Trial registration:**

ClinicalTrials.gov, NCT02058888. Registered February 10 2014

**Electronic supplementary material:**

The online version of this article (10.1186/s12915-019-0692-y) contains supplementary material, which is available to authorized users.

## Background

Healthcare-associated infections with antibiotic-resistant pathogens are increasing worldwide, posing a serious threat to our healthcare system [1, 2]. According to current estimates, up to ten million fatal cases are expected to be caused by antibiotic-resistant pathogens in 2050 [3].

In light of the emergence of novel sequencing techniques, we are now able to characterize the human microbiome and its associated resistome in detail. A major target for these investigations is the human gut because its microbiome is a well-known reservoir for a vast number of antibiotic resistance genes (ARGs) and moreover a hub for their horizontal exchange [4, 5]. It is likely that the human gut microbiome is a key player in the emergence and spread of antibiotic-resistant pathogens [6] and that its characterization can contribute to personalized antimicrobial stewardship (AWS) strategies.

Antibiotic treatment can have a massive impact on both the human gut microbiome and its resistome [7–9]. It is likely that the clinically most relevant antimicrobial selection pressure occurs in this ecosystem. Our group has previously reported on a methodology to determine the intestinal antimicrobial selection pressure under ciprofloxacin treatment using shotgun metagenomics [10]. The primary objective of our prospective, multicenter cohort study was to quantify and directly compare the antimicrobial selection pressure caused by ciprofloxacin or cotrimoxazole in a hematological patient population and to investigate how and to what degree individual patient characteristics and clinical cofactors influence the impact of antibiotics.

## Results

### Clinical cohort characteristics

We investigated two clinical cohorts from hematology departments in Tübingen and Cologne, Germany. Both cohorts received oral antibiotics as a prophylactic measure according to national clinical guidelines. In Tübingen, ciprofloxacin was administered, in Cologne cotrimoxazole. Ciprofloxacin belongs to the class of fluoroquinolone antibiotics. Cotrimoxazole contains two different substances which belong to different antibiotic classes. It consists of one part of trimethoprim, which blocks the bacterial folate metabolism, and of five parts of sulfamethoxazole, which belongs to the group of sulfanilamide antibiotics. We recruited 68 patients and included 41 into our final analysis. A flow chart of study participants and excluded patients is shown in Additional file 1: Figure S1.

Clinical and demographic characteristics are listed in Table 1. While most patient characteristics were similar in both cohorts, we identified differences in the underlying diseases (leukemia, lymphoma), laboratory parameters before start of antibiotic treatment (creatinine, bilirubin, platelet count), and concurrent medication (virostatic agents, antifungals).

**Table 1:** Major demographic and clinical characteristics of both treatment cohorts

Characteristic	Ciprofloxacin cohort (*n* = 20)	Cotrimoxazole cohort (*n* = 21)	*p* value
Demographic characteristics			
Female gender (%)	9 (42.9)	9 (45)	0.89
Age, years, mean	55.75	51.43	0.39
Weight, kg, median	80	78	0.24
Height, cm, mean	175.44	175.2	0.85
Persons in household, median	2	2	0.06
Underlying diseases and comorbidities			
Leukemia (%)	10 (50)	3 (14.3)	0.014*
Lymphomas (%)	8 (40)	19 (90.5)	0.001*
Charlson Comorbidity Score, median	2	2	0.51
Glascow Coma Scale, mean	15	15	n.a.
Baseline laboratory parameters			
Creatinine, mg/dl, median	0.75	0.87	0.004*
Bilirubin, mg/dl, mean	0.65	0.48	0.01*
Platelets, counts/μl, median	99,500	210,000	0.0003*
WBC, counts/μl, median	5790	7830	0.06
Neutrophils, counts/μl, median	2105	4130	0.09
Baseline medication and medication history			
Antibiotics within the last year (%)	3 (15)	4 (19)	0.73
Virostatic agents within the last year (%)	0 (0)	1 (4.8)	0.32
Anti-cancer treatment (%)	8 (40)	4 (19)	0.14
Virostatic agents (%)	7 (35)	1 (4.8)	0.015*
Antifungals (%)	6 (30)	0 (0)	0.007*
Proton pump inhibitor (%)	8 (40)	13 (61.9)	0.16
Cholesterol-lowering agents (%)	3 (15)	0 (0)	0.07
Bowel movement regulators (%)	6 (30)	4 (19)	0.41
Baseline microbiome/resistome/plasmidome parameters			
Microbiome Shannon diversity (phylum)	0.751	0.808	0.32
Microbiome evenness (phylum)	0.0115	0.012	0.19
Microbiome Shannon diversity (species)	4.51	4.55	0.81
Microbiome evenness (species)	0.0022	0.0018	0.26
Total plasmid abundance (coverage/10^6^ reads)	875.77	1066.92	0.06
Proteobacteria plasmid abundance (coverage/10^6^ reads)	172.43	193.82	0.14
Total plasmid Shannon diversity	6.18	6.52	0.24
Total plasmid evenness	0.0225	0.0227	0.75
Resistome Shannon diversity	1.0048	1.0639	0.24
Resistome evenness	0.3573	0.3612	0.40

*Statistically significant differences

*WBC* white blood cells

Stool samples were collected before treatment (T0, from now on called “baseline”), day 1 (T1), day 3 (T2) after initiation of antibiotic treatment, and at the end of the observation period (T3), which was after a median of 6 days on antibiotic treatment. Shotgun metagenomics was performed at each time point, with a median sequencing depth of 83,345,082 raw sequence reads per sample and 82,616,415 sequence reads per sample after filtration (about 12.39 Gb output). Microbiome, resistome, and plasmidome parameters at baseline did not differ between both treatment cohorts (Table 1).

The mean time period between hospital admission and collection of the baseline stool sample (with a subsequent start of antibiotic treatment) was 1.95 days in the ciprofloxacin cohort (range 0–6 days) and 1.47 days in the cotrimoxazole cohort (range 0–7 days) (Additional file 2: Table S1). We did not detect a statistical difference between both cohorts regarding time to baseline stool sample (*p* = 0.37). This data shows that our patients have received prophylactic antibiotic treatment shortly after hospital admission. We have chosen to investigate hematological cohorts with high-risk patients because the majority of these patients received antibiotics early during the hospital stay and since antibiotic resistance is a significant problem in this patient population. This is also the reason why we did not recruit a cohort of patients not treated with antibiotics as controls. Such a cohort is difficult to establish and would significantly differ from hematological patients in need of prophylactic or therapeutic antibiotic treatment.

### Impact of antibiotic treatment on the gut microbiome

**Fig. 1.**
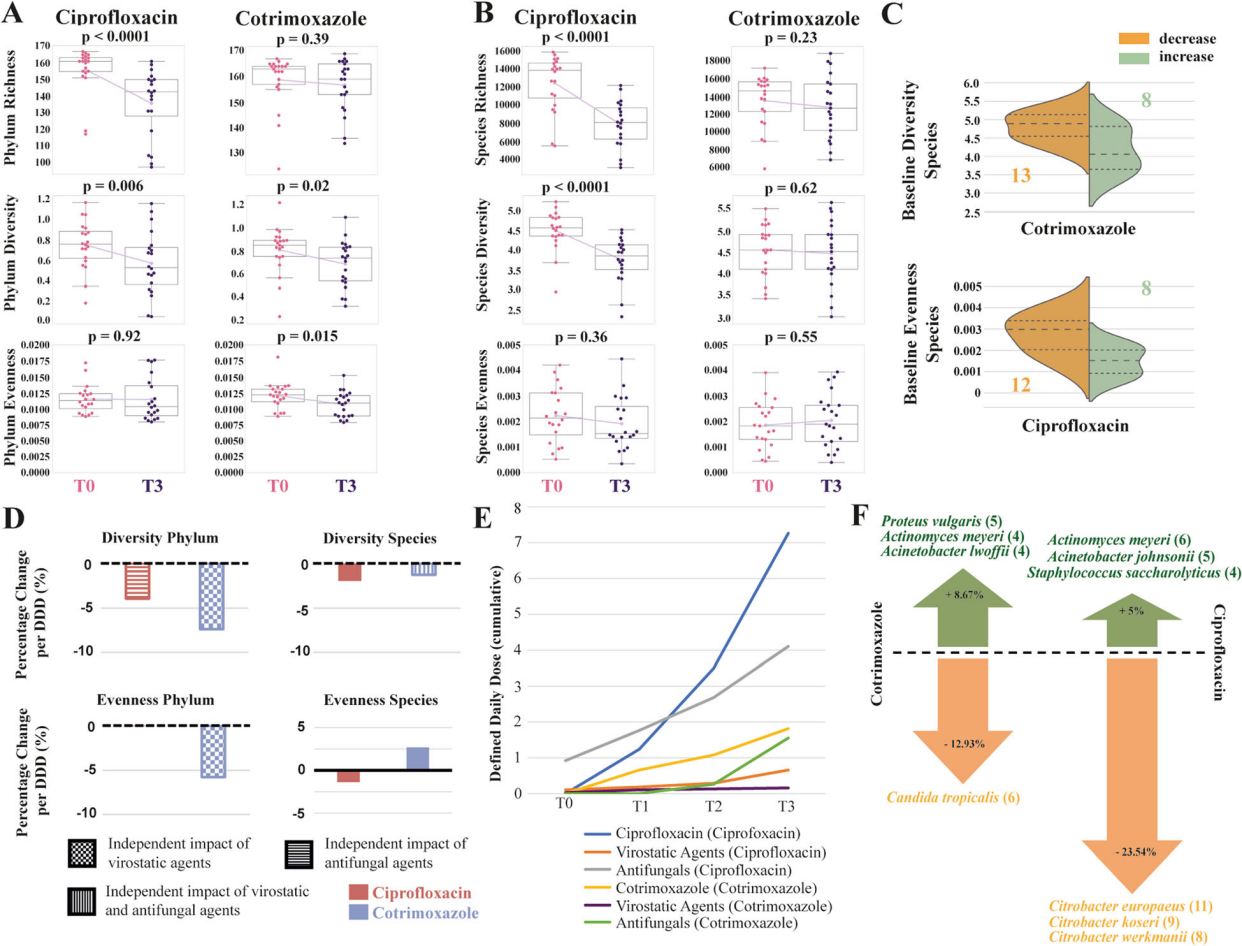
Antibiotic impact on the gut microbiome. Trajectories of richness, Shannon diversity, and Simpson’s evenness before treatment (T0) and at the end of the observation period (T3) are shown on phylum rank (**a**) and species rank (**b**) for both antibiotic treatments. Pink data points are measurements at T0, purple data points at T3. Boxplots indicate the distribution of data. The connecting magenta line shows the means at each time point and their development under treatment. The *p* value is displayed at the top of each box and indicates statistical significant differences between T0 and T3 within each treatment cohort (paired *t*-test). Under ciprofloxacin treatment, richness and Shannon diversity decrease significantly while Simpson’s evenness remains stable. In contrast, under cotrimoxazole, loss of richness and diversity is less pronounced and only significant on the phylum rank. **c** Violin plots illustrate the differences in baseline values between those patients with a positive baseline-endpoint comparison (BEC, green color) and those with a negative (orange color). The group size is displayed in the respective colors. Baseline species Shannon diversity was higher in the group of patients that lost diversity under cotrimoxazole, while patients with no decline or even an increase in diversity had a lower baseline diversity. The same was observed for species Simpson’s evenness under ciprofloxacin. **d** Based on multivariate regression modeling, the average percentage change per defined daily dose (DDD) is illustrated for each treatment cohort. Under both antibiotics, a loss in diversity was observed. However, no statistically significant difference was detected between both antibiotics. If an additional impact of concurrent medication was detected beside antibiotics in the multivariate models, this has been illustrated by different filling pattern. **e** Mean cumulative dose for antimicrobial agents in DDDs for the ciprofloxacin cohort and the cotrimoxazole cohort at each sampling time point (T0–T3). The colors indicate the drug classes, administered in either the ciprofloxacin or cotrimoxazole cohort (illustrated in brackets). The cumulative dose of ciprofloxacin was higher than the dose of cotrimoxazole. **f** Mean emergence and disappearance of species under antibiotic treatment in percentage compared to the species count at baseline. Frequent potentially pathogenic species are displayed. The number of patients with an emergence or disappearance of these species is shown in brackets

In both cohorts, we compared the impact of two prophylactic regimens on microbial richness, Shannon diversity and Simpson’s evenness of the gut microbiome (Fig. 1). At first, we investigated a crude baseline-endpoint comparison (BEC), where we compared differences between the last observation point (T3) and the baseline (T0, before treatment), following the equation BEC = variable (T3) − variable (T0). This way, BEC detects either an increase or decrease of the investigated variable over the course of antibiotic treatment. Hence, BEC reflects crude study results without considering differences in dosage or contributing factors.

We observed in both treatment cohorts a decline in Shannon diversity at a phylum level over the course of the treatment (Fig. 1a). However, the mean decline was greater under ciprofloxacin treatment (− 31.29%, *p* = 0.006) compared to cotrimoxazole (− 17.95%, *p* = 0.02). On a species level (Fig. 1b), we only observed a mean decline under ciprofloxacin (− 21.01%, *p* <  0.0001) but not under cotrimoxazole (− 2.01%, *p* = 0.62). The chance of whether diversity decreased or increased in a patient was dependent on the baseline status in the cotrimoxazole cohort (Fig. 1c). Patients with a high baseline diversity were more likely to lose diversity, while those with a lower baseline diversity likely remained at the same level or even slightly gained diversity when treated with cotrimoxazole (*p* = 0.01, Additional file 3: Table S2).

Evenness showed a statistically significant decrease on the phylum level for patients on cotrimoxazole (− 13.2%, *p* = 0.015), indicating some disruption of the original phylum composition. On the species level, we did not note a decrease in evenness on both antibiotics (Fig. 1a, b). Alteration in evenness was found to be dependent on the evenness baseline status in the ciprofloxacin cohort (Fig. 1c, Additional file 3: Table S2, *p* = 0.006). All baseline disparities are presented in Additional file 3: Table S2.

We also computed multivariate regression models which can handle the entire time series data (T0, T1, T2, and T3) of all patients. Instead of just investigating the crude study outcome (BEC analysis), this furthermore enabled us to take different cumulative dosages of antibiotics into account and to test for the contributing effect of all variables that had turned out to be significantly different between both treatment cohorts (Table 1). We also included proton pump inhibitors into this analysis since their influence on the microbiome has been previously reported [11, 12].

This investigation was independently done for both cohorts. It started with a univariate regression analysis of the antibiotic effect for each outcome variable and a subsequent analysis of potential contributing variables. If contributing variables were detected (*p* <  0.05), they were included into the model with the antibiotic, resulting in a multivariate model with adjusted model coefficients. Finally, potential differences between the trends (regression coefficients) of both antibiotics for a certain outcome variable (e.g., Shannon diversity) were investigated using the likelihood ratio test (LR). Within each regression model, data from one patient was treated as its own time series within the model by data clustering. This resulted in the effect that each patient served as its own control by comparing the baseline with the subsequent time points. Finally, the model itself reports an overall effect for the cohort.

Figure 1d demonstrates normalized multivariate regression coefficients indicating an average percentage change of diversity/evenness per cumulative defined daily dose (DDD) of the antibiotic. Overall, the results show a decrease of diversity in both cohorts. For evenness, a decrease was noted on phylum and an increase on species level for patients on cotrimoxazole prophylaxis. Evenness was just slightly impacted in the ciprofloxacin group. Along with antibiotic treatment, other factors including baseline creatinine, lymphoma as underlying disease, and virostatic/antifungal treatment also had a significant impact on intestinal microbiome diversity and evenness when tested in the multivariate models (Fig. 1d, Additional file 4: Table S3). Crude results of the univariate models are displayed in Additional file 5: Table S4.

Interestingly, after accounting for the different cumulative antibiotic dosages and these cofactors, we did not observe statistical significant differences in antimicrobial selection pressure caused by both antibiotics (LR *p* ≥ 0.18 for all microbiome variables, Additional file 4: Table S3), suggesting both antibiotics have a similar effect on the microbiome. The BEC analysis had indicated such differences (Fig. 1a, b). But they clearly do not exist after multivariate adjustment, suggesting a strong impact of the identified cofactors.

One major factor that impacts the results of the multivariate modeling is the different mean cumulative dose of antibiotics that both groups received (Fig. 1e). Ciprofloxacin was administered at about fourfold higher cumulative doses when compared with cotrimoxazole. Multivariate modeling takes this into account, while BEC does not. Trajectories of microbiome variables over all time points and for all patients are shown in Additional file 6: Figure S2 and Additional file 7: Figure S3.

The drop in microbiome diversity on antibiotic treatment came also along with a disappearance of microbial species (Fig. 1f). However, compared to the baseline species count, we also observed an emergence of species not detectable before treatment (8.67% on cotrimoxazole and 5% on ciprofloxacin, respectively). Among the emerging species were potential pathogens like *Proteus vulgaris* and *Acinetobacter lwoffii/johnsonii*, illustrating important shifts during antibiotic treatment.

### Impact of antibiotic treatment on the gut resistome

Within our study, we also set out to determine the influence of antimicrobial therapy on the gut resistome. Therefore, the sequencing reads were mapped to the ARG-ANNOT resistance gene database [13]. A total of 382 ARGs belonging to different ARG classes have been detected in all samples.

**Fig. 2.**
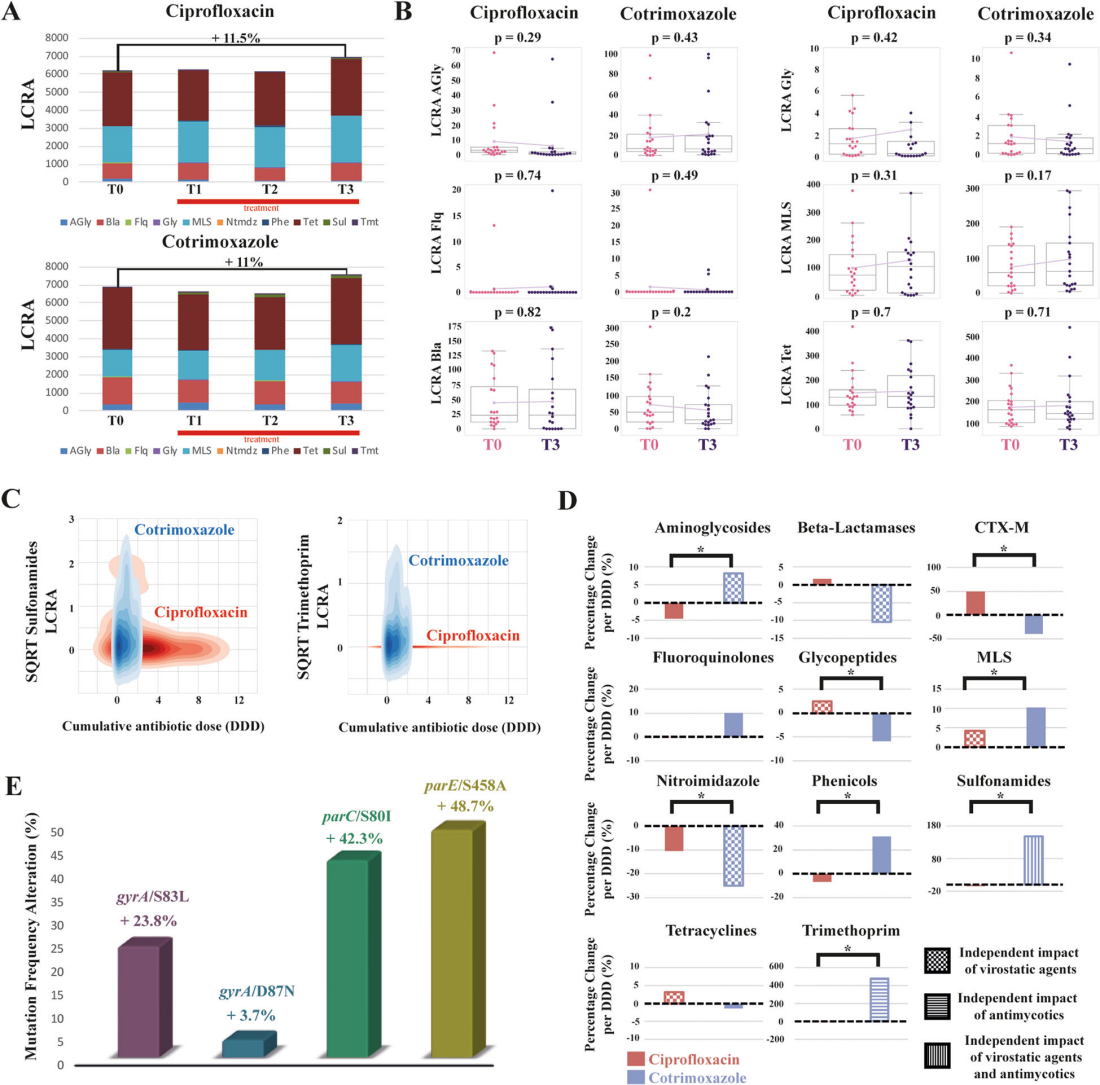
Antibiotic impact on the gut resistome. **a** Stacked bar chart of summed length-corrected relative abundances (LCRA) of major antimicrobial resistance gene (ARG) classes at baseline (T0) and over the treatment period (T1–T3). The following ARG classes are depicted: aminoglycosides (AGly), beta-lactamases (Bla), fluoroquinolones (Flq), glycopeptides (Gly), macrolide-lincosamide-streptogramin (MLS), nitroimidazoles (Ntmdz), phenicols (Phe), sulfonamides (Sul), tetracyclines (tet), and trimethoprim (Tmt). **b** Trajectories of antimicrobial resistance genes quantification by LCRA before treatment (T0) and at the end of the observation period (T3) are shown for both antibiotic treatments. Pink data points are measurements at T0, purple data points at T3. Boxplots indicate the distribution of data. The connecting magenta line shows the means at each time point and their development under treatment. The *p* value is displayed at the top of each box and indicates statistical significant differences between T0 and T3 within each treatment cohort (paired *t*-test). Trends for LCRA changes are prominent but do not reach statistical significance. **c** Two-dimensional kernel estimation density of square root transformed LCRA values of sulfonamide and trimethoprim ARG classes in relation to the administered cumulative antibiotic dose in defined daily doses (DDD). ARG LCRA rises significantly with increasing doses of cotrimoxazole, but not under ciprofloxacin. **d** Based on multivariate regression modeling, the average percentage change of ARG class LCRA per defined daily dose (DDD) is illustrated for each treatment cohort. Bonferroni-corrected statistically significant differences between both antibiotics (LR *p* < 0.002) are presented by single asterisks. Significant differences in antimicrobial selection pressure were observed for aminoglycoside, CTX-M, glycopeptide, MLS, nitroimidazole, phenicol, sulfonamide, and trimethoprim ARGs. If an additional impact of concurrent medication was detected beside antibiotics in the multivariate models, this has been illustrated by different filling pattern. **e** Fluoroquinolone resistance-mediating mutation frequencies increase under ciprofloxacin exposure in patient 512 comparing baseline (T0) and endpoint (T3)

The total length-corrected relative abundance (LCRA) of the most abundant ARG classes did surprisingly increase just by 11.5% (*p* = 0.43) on ciprofloxacin and 11% (*p* = 0.55) on cotrimoxazole between the baseline and end of treatment (Fig. 2a). Hence, the total ARG LCRA did not significantly change over the treatment period.

We also investigated LCRA shifts of single ARG classes for both antibiotics using BEC (Fig. 2b). As for the total ARG LCRA, none of these BEC shifts were statistically significant. However, we observed a fairly strong mean increase of sulfonamide (+ 354.4%, *p* = 0.07) and trimethoprim (+ 894.4%, *p* = 0.14) ARGs under cotrimoxazole (Fig. 2c) compared to low BEC values for sulfonamide (+ 3.8%, *p* = 0.93) and trimethoprim (+ 6.25%, *p* = 0.96) ARGs under ciprofloxacin. This suggests differences between both antibiotic treatments. BEC results for all observed ARG classes are shown in Additional file 8: Figure S4. LCRA trajectories of all ARG classes comprising all patients and sample time points are shown in Additional file 9: Figure S5, Additional file 10: Figure S6, Additional file 11: Figure S7 and Additional file 12: Figure S8.

A potential reason for the high variance in ARG LCRA observed in BEC could be that the impact of antibiotic treatment on the intestinal resistome is very patient specific, for instance depending on the individual microbiome and resistome baseline status and also on other individual patient characteristics. By applying multivariate regression modeling, we additionally investigated potential cofactors and corrected for differences in the cumulative antibiotic dosage (Fig. 2d, Table 2, Additional file 13: Table S5). Confirming the BEC analysis, we observed a high antimicrobial selection pressure for sulfonamide and trimethoprim ARGs, which increased per cumulative cotrimoxazole DDD by 148.1% and 477.7% (*p* = 0.015 and *p* = 0.1), respectively. Crude results of the univariate models for ARGs are displayed in Additional file 14: Table S6.

**Table 2:** Multivariate selection pressure estimates for major antibiotic resistance gene classes

ARG class	Ciprofloxacin coefficient	Ciprofloxacin coefficient (normalized)	Cotrimoxazole coefficient	Cotrimoxazole coefficient (normalized)	LR *p* value (likelihood ratio test)
Aminoglycoside ARGs	− 0.4	− 4.44%	1.45	8.16%	< 0.0001
Beta-lactamases	0.73	1.63%	− 7.44	− 10.39%	0.04
CTX-M	0.05	50%	− 0.02	− 40%	< 0.0001
Fluoroquinolone ARGs	− 0.001	− 0.14%	0.15	10.06%	0.79
Glycopeptide ARGs	0.04	2.48%	− 0.11	− 5.88%	< 0.0001
MLS ARGs	4.22	4.23%	7.76	10.25%	< 0.0001
Nitroimidazole ARGs	− 0.0008	− 10.52%	− 0.006	− 25%	< 0.0001
Phenicol ARGs	− 0.06	− 6.52%	0.56	31.11%	< 0.0001
Sulfonamide ARGs	− 0.13	− 5.03%	2.34	148.1%	< 0.0001
Tetracycline ARGs	4.57	3.05%	− 2.52	− 1.45%	0.94
Trimethoprim ARGs	− 0.004	− 2.5%	0.86	477.77%	< 0.0001

*ARG* antibiotic resistance gene, *CTX-M* plasmid-mediated cefotaximases, *MLS* macrolide-lincosamide-streptogramin

Normalized coefficients are based on the mean baseline ARG length-corrected relative abundance (LCRA) and demonstrate a relative change in ARG LCRA per defined daily dose (DDD) of the antibiotic

Particularly interesting was the comparison of antimicrobial selection pressure from all ARG classes between both antibiotic treatments using the likelihood ratio test (LR). This revealed significant differences in antimicrobial selection pressure for various ARG classes which are of clinical relevance (Fig. 2d, Table 2). For instance, we observed a positive selection pressure for CTX-M with ciprofloxacin, while negative with cotrimoxazole (LR *p* <  0.0001). Additionally, we observed a high positive selection pressure for sulfonamide and trimethoprim ARGs under cotrimoxazole as mentioned above. This was clearly not the case under ciprofloxacin treatment (LR *p* <  0.0001 for both), suggesting that ARGs conferring antimicrobial resistance to the substances contained in cotrimoxazole expand exclusively under the respective treatment.

The suspicion that the high variance in ARG LCRA observed in BEC could be driven by individual cofactors that differ between patients was confirmed in our multivariate analysis. Similar to microbiome changes, several cofactors like bilirubin, creatinine, underlying hematological diseases, proton pump inhibitors and mostly concurrent antimicrobial agents independently shaped ARG LCRA under antibiotic treatment (Additional file 13: Table S5). This contributing effect was particularly pronounced for virostatic agents, which had a significant impact on ARG LCRA in 7 of 11 ARG classes, thus appearing to be a driving force of resistome alterations.

### Impact of ciprofloxacin on the length-corrected relative abundance of fluoroquinolone ARGs and resistance-mediating mutations

Overall, we did not observe differences between the two antibiotics with respect to selection of fluoroquinolone ARGs (Fig. 2b, d). In ARG-ANNOT [13], this ARG class includes *qnr* genes and efflux pumps. We only detected *qnr* genes in our dataset. Since we observed a low frequency of *qnr* genes in our cohort (Additional file 9: Figure S5), it is difficult to compute antimicrobial selection pressure differences between both drugs.

We therefore additionally examined our cohorts for the presence of common fluoroquinolone resistance-mediating mutations (*gyrA*, *parC*, *parE*, *acrR*, *acrB*) [14] using reference genes from *Escherichia coli* strain K-12 MG1655 and *Staphylococcus aureus* NCTC8225 and NCTC8325. We found four mutations mapping to the reference *E. coli* strain K-12 in one patient (ID 512) from the ciprofloxacin cohort. Figure 2e shows the percentage increase of sequence reads carrying the respective mutations comparing baseline (T0) and endpoint (T3). These results indicate a clear positive selection when fluoroquinolone resistance-mediating mutations are abundant before treatment. Patient 512 was the only one with such mutations at baseline. We also did not observe the emergence of sequence reads with fluoroquinolone resistance-mediating mutations under ciprofloxacin treatment in any patient.

The same patient (ID 512) also possessed *qnr* genes before ciprofloxacin administration, which significantly expanded on the first day of treatment but declined afterwards even to a state much lower than at baseline (Additional file 9: Figure S5). The non-linear course of resistome changes in this patient demonstrates the importance of the baseline status and the individual aspects of ARG selection.

As a consequence, we examined the overall influence of the baseline resistome status (T0) on the selection of ARG classes. Mean baseline ARG class LCRAs were compared from patients whose ARG class LCRAs increased or decreased over the observation period (BEC values). We found that baseline ARG class LCRAs were significantly relevant for antimicrobial selection in four ARG classes (Additional file 15: Table S7). This included aminoglycoside and CTX-M ARGs in the ciprofloxacin cohort. For aminoglycoside ARGs, high baseline LCRA was likely to decrease during treatment, while high CTX-M baseline LCRA was likely to increase. For cotrimoxazole, high baseline LCRA levels of glycopeptide and phenicol ARGs were more likely to result in a decrease of these ARGs. This demonstrates that resistome alteration under antibiotic treatment depends on the baseline resistome, but only for specific ARG classes. Quantitative LCRA baseline levels for all ARG classes and both treatment cohorts can be found in Additional file 15: Table S7.

### Localization of ARGs

Our results indicate that different antibiotics have a specific effect on the gut resistome. However, determining distinctions between antibiotics relating to antimicrobial selection pressure adjusted to certain cofactors is just one first step in improving antibiotic treatment strategies. Additionally, the clinical relevance of an ARG or ARG class must be a vital element in the overall decision-making process in how to administer antibiotics. Clinical relevance of an ARG (class) is determined (i) by the importance of the antibiotic class that is rendered useless by an ARG, (ii) by the taxonomic unit carrying the ARG, and (iii) by the genomic location of the ARG, particularly whether or not it is located on a mobile genetic element.

For these reasons, we also investigated the taxonomic location of ARG classes in our patients. ARGs are primarily an immediate threat to patients when they are carried by pathogenic organisms. We established a Kendall’s rank correlation network between taxonomic phyla and ARG classes for the ciprofloxacin (Additional file 16: Figure S9A) and the cotrimoxazole cohort (Additional file 16: Figure S9B) over all observation time points.

In the ciprofloxacin cohort, glycopeptide resistance-mediating ARGs including *van* genes were associated with a location in the phylum Firmicutes (tau correlation coefficient = 0.37, *p* = 1.6 × 10^− 6^). Firmicutes comprise the genus Enterococci, which are increasingly found to be vancomycin resistant [15].

In the cotrimoxazole cohort, sulfonamide and trimethoprim ARGs were associated with Proteobacteria (tau = 0.15, *p* = 0.06 and tau = 0.23, *p* = 0.004, respectively), while fluoroquinolone ARGs were associated with Proteobacteria in the cotrimoxazole and ciprofloxacin cohort (tau = 0.2, *p* = 0.017 and tau = 0.37, *p* <  0.00003, respectively).

Since the phylum Proteobacteria contains several clinically important pathogens, we extended our correlation network to the species level (Additional file 17: Table S8). In the cotrimoxazole cohort, we found potentially pathogenic *Enterobacter* sp., *Citrobacter* sp., *Klebsiella* sp., and *Serratia marcescens* to be positively correlated with sulfonamide and trimethoprim ARGs, suggesting some degree of pathogen selection under cotrimoxazole treatment. We also found positive correlation in the ciprofloxacin cohort. *Escherichia coli*, *Citrobacter* sp., *Enterobacter cloacae*, *Serratia marcescens*, *Staphylococcus aureus*, and *Staphylococcus saccharolyticus* were positively correlated with fluoroquinolone ARGs.

### Impact of antibiotic treatment on the intestinal plasmidome

While an ARG location in an apathogenic commensal organism might not pose an immediate threat to a patient, it could be a future threat if the ARG is localized on a mobile genetic element. Therefore, we investigated how the plasmidome is driven by antibiotic treatment and to what extent it is involved in the expansion of ARGs.

**Fig. 3.**
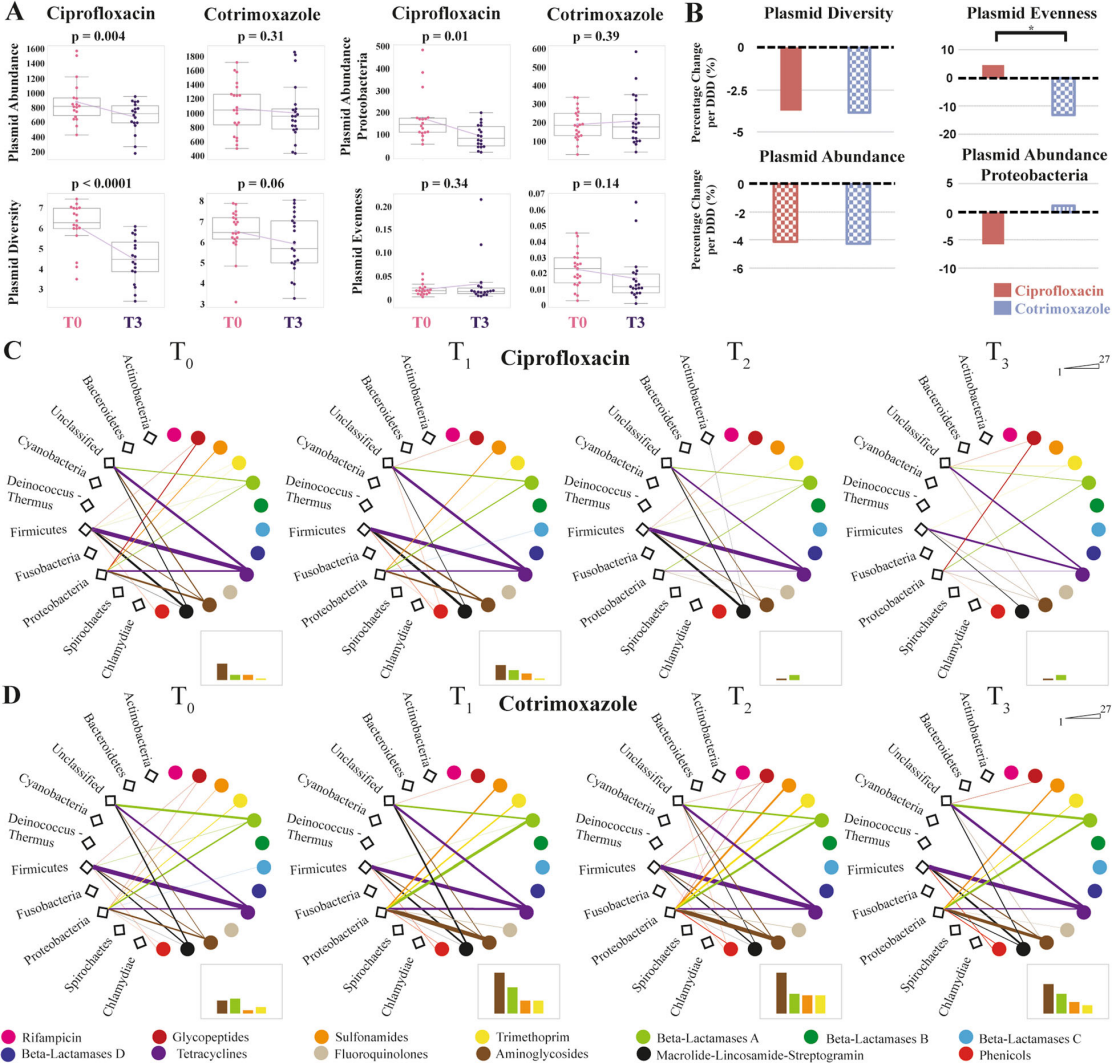
Antibiotic impact on the gut plasmidome. **a** Trajectories of total plasmid abundance, plasmid abundance from proteobacteria, plasmid Shannon diversity, and plasmid Simpson’s evenness before treatment (T0) and at the end of the observation period (T3) are shown for both antibiotic treatments. Pink data points are measurements at T0, purple data points at T3. Boxplots indicate the distribution of data. The connecting magenta line shows the means at each time point and their development under treatment. The *p* value is displayed at the top of each box and indicates statistical significant differences between T0 and T3 within each treatment cohort (paired *t*-test). Total plasmid abundance, plasmid abundance from Proteobacteria, and plasmid diversity decreased significantly under ciprofloxacin treatment while plasmid evenness remained stable. In contrast, plasmids were not strongly affected by cotrimoxazole. **b** Based on multivariate regression modeling, the average percentage change of plasmid characteristics per defined daily dose (DDD) is illustrated for each treatment cohort. Bonferroni-corrected statistically significant differences between both antibiotics (LR *p* < 0.002) are presented by single asterisks. If an additional impact of concurrent medication was detected beside antibiotics in the multivariate models, this has been illustrated by a different filling pattern (checkerboard pattern = virostatic agents, horizontal stripes = antifungal agents, vertical stripes = virostatic and antifungal agents). Trends for plasmid evenness were significantly different, with a slight increase under ciprofloxacin and moderate decrease under cotrimoxazole. **c**, **d** The co-occurrence network displays the relationship between ARG-carrying plasmids from certain taxonomic origins and the ARG classes located on these plasmids at each sample collection time point for the ciprofloxacin cohort (**c**) and the cotrimoxazole cohort (**d**). The total plasmid-ARG content is expressed by the line width between plasmid origin and ARG class. The bar on the upper right part of each network row displays the scale of the total plasmid-ARG content (range 1–27). The diagrams in the lower right parts illustrate the Proteobacteria plasmid-ARG content for aminoglycoside, sulfonamide, trimethoprim ARGs, and beta-lactamase A enzymes. The *y*-axis ranges from 1 to 27 and displays the respective plasmid-ARG content. The ARG classes in the diagrams correspond to the colors of the networks and the legend at the bottom of the graph. Plasmids harboring ARGs from Proteobacteria expanded under cotrimoxazole, while ARG-containing plasmids from all origins declined under ciprofloxacin

Comparing the last time point (T3) with the baseline (T0) in our study (BEC analysis), we observed a mean decrease in plasmid diversity (− 37.3%, *p* <  0.0001), total plasmid abundance (− 36.11%, *p* = 0.004), and plasmid abundance from Proteobacteria (− 87.6%, *p* = 0.01) under ciprofloxacin (Fig. 3a). Of note, plasmid diversity and abundance were not significantly affected by cotrimoxazole, although mean plasmid diversity decreased to some extent (− 10.13%, *p* = 0.06). Plasmid evenness remained stable on both treatments, though this depended on its baseline status (cotrimoxazole *p* = 0.05, ciprofloxacin *p* = 0.004, Additional file 18: Table S9). The other plasmid variables did not show disparities in their baseline status (Additional file 18: Table S9). The entire time series for plasmid variables is displayed in Additional file 19: Figure S10.

Multivariate regression modeling taking contributing factors and the different cumulative dosage into account demonstrated that plasmid diversity and total plasmid abundance declined to the same extent in both treatment groups (Fig. 3b, Additional file 20: Table S10). Plasmid evenness was significantly different between both antibiotic treatments (LR *p* <  0.0001), with a decrease under cotrimoxazole and a slight increase under ciprofloxacin. Additional file 21: Table S11 additionally displays the results from the univariate analysis.

As with BEC analysis, we examined the abundance from Proteobacteria plasmids separately, since many of them contain ARGs. The total plasmid abundance and abundance of plasmids from Proteobacteria significantly decreased under ciprofloxacin (both *p* = 0.002, Additional file 20: Table S10, Fig. 3a and b), but not under cotrimoxazole (*p* = 0.24 and *p* = 0.86, Additional file 20: Table S10, Fig. 3a). We did not note a clear differential impact between both drugs on plasmid abundances when considering a Bonferroni-corrected LR *p* value < 0.002 (Fig. 3b, Additional file 20: Table S10). Nevertheless, the determined LR *p* value of 0.02 still presents potential differences in the impact of both antibiotics on plasmid abundances, particularly in plasmids from Proteobacteria, with a decrease of plasmid abundance under ciprofloxacin and a stable state or even slight expansion under cotrimoxazole (Fig. 3b, Additional file 20: Table S10). The moderate decrease in plasmid evenness under cotrimoxazole pointed to the emergence of a sudden dominance of a few plasmids, likely because of positive selection (Fig. 3b, Additional file 20: Table S10).

We therefore addressed the question of whether plasmids from Proteobacteria or other taxonomic origins carried ARGs and were then selected in the patients’ guts during treatment. We computed a co-occurrence network that displays the relationship between the taxonomic origin of ARG-carrying plasmids and their total plasmid-ARG content for each time point of our study period and for both treatment cohorts (Fig. 3c for ciprofloxacin, Fig. 3d for cotrimoxazole). Under ciprofloxacin, we observed a decline in the total plasmid-ARG content over the course of treatment. This is presumably due to an extinction of species which carry plasmids with ARGs. Under cotrimoxazole on the other hand, we noticed a sudden increase of Proteobacteria-derived plasmids carrying sulfonamide, trimethoprim, aminoglycoside ARGs, and A-beta-lactamases. This suggests a rise in the abundance of ARG-carrying plasmids from a potentially pathogenic origin, providing evidence for a positive plasmid selection caused by cotrimoxazole.

Since horizontal gene transfer occurs more frequently between species from the same body site and phylogenetic background [16], this poses an additional threat regarding ARG transmission from one species to another, particularly considering the emergence of pathogenic species within the microbiome (Fig. 1f). The interplay between antibiotic treatment and plasmidome alteration that we observed was again independently influenced by other factors. These were mostly virostatic agents, particularly when combined with cotrimoxazole (Additional file 20: Table S10).

### Interplay between gut microbiome, resistome, and plasmidome under antibiotic pressure

Our results indicate that antibiotic selection is a non-linear process, depending on the presence and quality of cofactors. Understanding the complex interplay of these cofactors is important for implementing metagenomic-guided antimicrobial stewardship that by necessity integrates an exceptional high level of individuality.

**Fig. 4.**
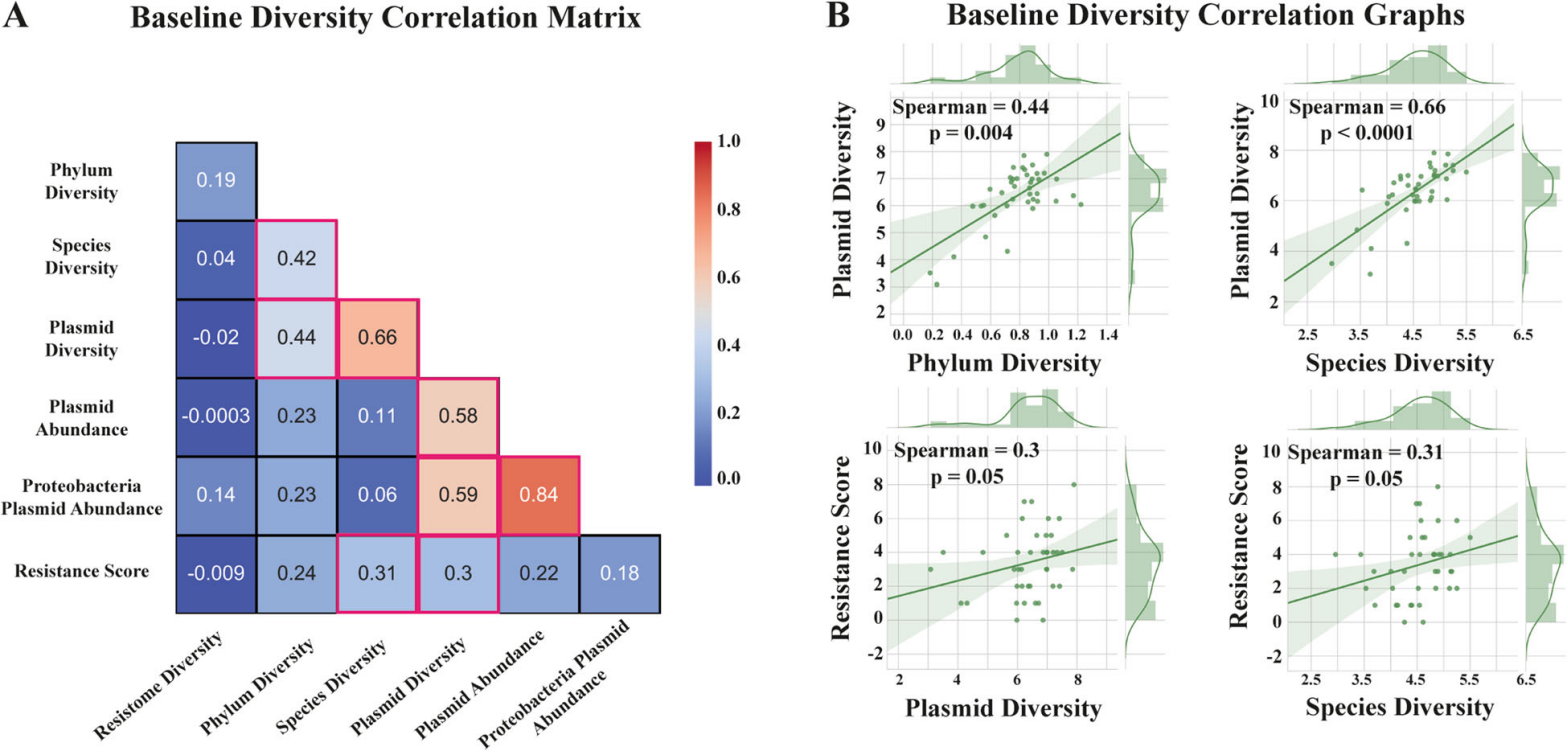
Links between baseline gut microbiome and resistome alteration under antibiotic pressure. **a** Spearman’s rank correlation matrix revealed a positive correlation between the resistance score (indicating more positive antibiotic resistance gene selection in patients) and baseline microbiome and plasmid diversity. Pink-colored edgings indicate statistically significant correlation coefficients (*p* ≤ 0.05). **b** Scatter graphs with detailed illustration of the relation between baseline microbiome and plasmid diversity as well as between resistance score and baseline microbiome and plasmid diversity

We created a correlation matrix composed of baseline taxonomic diversity and diversity of mobile genetic elements (Fig. 4a). We also created a resistance score for each patient on the basis of comparing ARG LCRA of the baseline (T0) with the end of the observation period (T3), again applying BEC. If a patient had experienced an increase in one of the ARG classes at the end of the observation period, this was scored with one point. The higher the score, the more we observed positive selection for more ARG classes.

The correlation matrix revealed that microbiome species diversity at baseline was positively correlated with the resistance score (rho = 0.31, *p* = 0.05). Thus, patients were more likely to present an increase in ARG LCRA while on treatment when baseline species diversity was high (Fig. 4a, b). It is important to note that there was also a strong correlation between baseline plasmid diversity and resistance score (rho = 0.3, *p* = 0.05, Fig. 4a, b). This could reflect a higher baseline potential for horizontal gene transfer resulting in a more effective ARG expansion. A subgroup analysis of both cohorts revealed that a correlation of resistance score with baseline plasmid diversity was specifically the case in the cotrimoxazole cohort (rho = 0.41, *p* = 0.04) and was weaker in the ciprofloxacin cohort (rho = 0.18, *p* = 0.45). Generally, baseline species diversity was highly correlated with baseline plasmid diversity (rho = 0.66, *p* <  0.001, Fig. 4a, b). In order to exclude confounding because of differences in the cumulative antibiotic dose of patients, we correlated this dose with the resistance score. We did not determine a relevant association (rho = 0.08, *p* = 0.64).

These findings indicate that high species diversity could indeed be a risk factor for an effective selection and spread of ARGs, underlining the importance of the microbiome baseline status before antibiotic treatment.

**Fig. 5.**
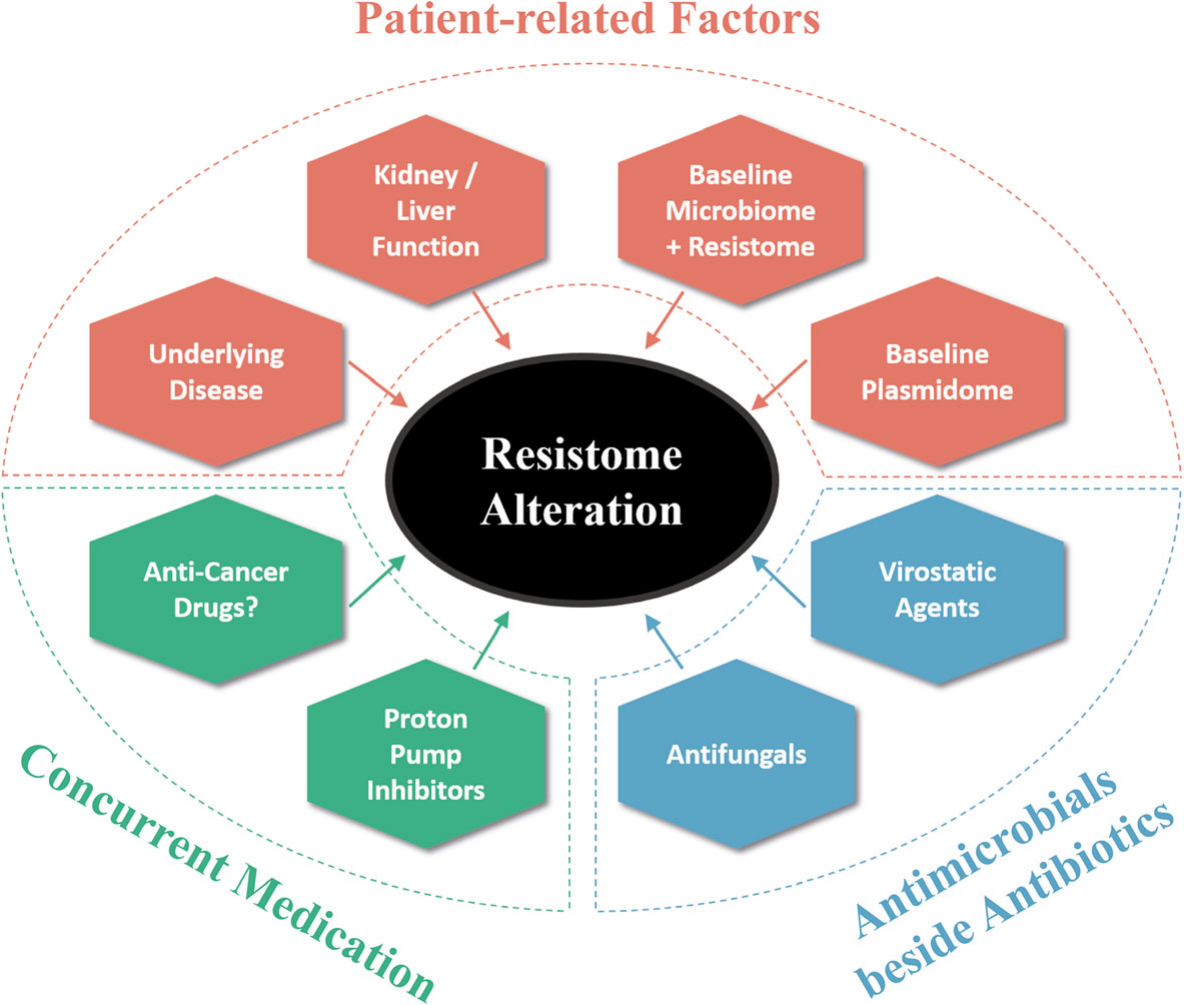
Independent contributors that shape the gut resistome along with antibiotic treatment. The graph summarizes the concept of additional independent variables that impact the alterations of the gut resistome under antimicrobial selection pressure caused by antibiotic treatment

We concluded that resistome alteration under the significant impact of antibiotic treatment is additionally driven by a complex interplay of various cofactors, including the baseline microbiome, resistome, and plasmidome, but also other individual patient factors like the liver and kidney function, and clinical cofactors like concurrent drugs, particularly virostatic agents (Fig. 5).

## Discussion

Our study presents the first proof-of-concept that ultra-deep shotgun metagenomics allows us to determine and compare antimicrobial selection pressure for different antibiotics in a clinical cohort of hematological patients. We compared ciprofloxacin with cotrimoxazole. While both antibiotics had a similar negative impact on gut microbiome diversity, there were significant differences in resistome alterations under treatment. Nevertheless, currently, it is not an option to generally recommend one of these antibiotics over the other regarding spread of resistance within a patient or between patients. In terms of resistome alterations, we discovered a complex interplay between the antibiotics with concomitant treatment, the clinical status of a patient, and the baseline status of the gut microbiome, resistome, and plasmidome. Of note, the microbiome, resistome, and plasmidome parameters at baseline were not different between both treatment cohorts. This makes a general bias regarding the microbiome baseline composition of our cohorts unlikely and emphasizes the validity of our observations. It is thus important to account for all identified contributors when predicting the impact of an antibiotic on resistome alterations of an individual patient. However, we cannot warrant that all relevant contributing patient and environmental variables were documented in our study and subsequently included into our final models, e.g., potential differences in the diet between both cohorts were not investigated.

In this context, we want to stress the contributing effect of virostatic agents. It has recently been reported that an unexpectedly high number of drugs affect microbiota, even those without a direct antimicrobial activity like proton pump inhibitors, antidiabetics, psychotropic drugs, and many more [17–19]. To our knowledge, however, this is the first study to show a relevant and independent impact of antiviral treatment on the microbiome, resistome, and plasmidome in a clinical cohort. We have also noted independent effects of antifungals and proton pump inhibitors, but to a lower extent. This is a clinically highly relevant finding since it has been reported that a diminished microbiome diversity—regardless of its cause—has a negative impact on long-term survival, particularly in patients with hematological malignancies [20, 21].

Baseline laboratory parameters like creatinine and bilirubin were further independent contributors, probably due to their importance for the pharmacokinetics of drugs or due to the interplay between liver metabolism and the gut microbiome [22]. For instance, increasing serum levels of creatinine shifted the resistome always in the same direction as cotrimoxazole in our study, probably due to the renal excretion of both drug’s components and their accumulation under reduced kidney function resulting in a prolonged effect.

Underlying hematological diseases were also identified as other important cofactors. We hypothesize that this might reflect the distinct anti-cancer treatments within the cohort because anti-cancer drugs have been reported to affect gut microbiota composition [18, 23]. Since anti-cancer treatment regimens were highly diverse in our cohorts, and since our study was specifically designed to investigate and compare the effect of antibiotics, we cannot provide further evidence regarding anti-cancer drugs as contributors. Instead, we recommend independent studies to specifically address this question.

We also found the baseline plasmidome to be one of the major players in rendering how an antibiotic would impact a patient’s resistome. In our clinical cohorts, we showed that high gut plasmid diversity before treatment reflects a higher transmission potential, and thus a higher chance for positive ARG selection under antibiotic pressure. On the other hand, antibiotic pressure can shape the plasmidome to a relevant degree. We saw a relative expansion of ARG-carrying plasmids from Proteobacteria under cotrimoxazole. The higher impact on the plasmidome compared with ciprofloxacin might be due to the selection of sulfonamide ARGs which are often localized on integron cassettes, typically to be found on conjugative plasmids [24].

One limitation of our study is the lack of a cohort not treated with antibiotics as a control. Abeles et al. have shown that relative abundances of bacterial taxa change over time in a similar pattern in household members either treated with an antibiotic or a placebo [25]. This indicates that various environmental contributing factors could have an impact on the microbiome beside antibiotics. In our study, we did not recruit a non-antibiotic-treated control cohort since we assumed this cohort would severely differ from hematological patients in need of antibiotics. Hence, we could have missed additional contributing factors from the hospital environment that further shape the microbiome, resistome, and plasmidome. We have potentially weakened this limitation by clustering the time series data from each patient within our regression models. This way, we have made each patient his or her own control. Moreover, the results of our clinical study stress some points regarding confounding: We must acknowledge individual contributing factors from patients if we want to determine the impact of antibiotic treatment with a high precision. And while we do not expect a strong “household effect” in the clinic due to the relatively short stay, we want to emphasize that studies are warranted which investigate the additional contribution of the hospital environment on microbiome, resistome, and plasmidome changes. This will enable to determine antibiotic impact even more precisely.

## Conclusions

Our study is one important exploration towards a metagenomic-guided antimicrobial stewardship that aims at advanced and informed precision for the use of antibiotics in a high-risk hematological patient population. Predicting the individual effect of an antibiotic seems possible, but this will need to incorporate multiple contributors in order to completely reflect the complex interplays outlined by our data. A profound knowledge of these cofactors will enable us to collect required data in an appropriate format in large cohorts and to measure the specific impact on clinically relevant resistome partitions. A link between significant resistome-shaping factors with clinically relevant selection of resistance could subsequently be modeled through machine-learning algorithms for predicting the effects of individual antibiotic treatments and for supplying therapeutic advice. Such computer-supported individualized guidance would not only promote the transition of infectious disease medicine into the digital age, but also provide the means to significantly reduce transmission of resistant pathogens, thus improving infection control and patient safety.

## Methods

### Hospital settings

We conducted a prospective, multicenter cohort study at two university hospitals in Tübingen and Cologne, Germany, in order to assess the impact of antibiotic treatment on the gut resistome and to compare antimicrobial selection pressure between different antibiotic prophylaxis regimens. In both hospitals, patients were recruited from the hematology/oncology departments. The local ethics committees approved the study (reference numbers: 661/2013BO1 and 14-021, respectively). All patients provided written informed consent before participating in the study. Data monitoring of patient data was performed at both centers. The study is registered at https://www.clinicaltrials.gov/ under the identifier NCT02058888.

### Study design, definition, and participants

Adult patients (≥ 18 years) with an underlying hematological-oncological disease were considered eligible if a neutropenia of at least 7 days and the need for an antibiotic prophylaxis were expected. Patients having received antibiotics within the last 30 days were excluded from the study. A complete list of inclusion and exclusion criteria is made available in Additional file 22: Table S12. Patients in Tübingen received oral ciprofloxacin as prophylaxis against bacterial infections during neutropenia (2 × 500 mg daily), patients in Cologne oral cotrimoxazole (trimethoprim/sulfamethoxazole) as *Pneumocystis jirovecii* pneumonia prophylaxis (160/800 mg three times a week). Patients were excluded from the study if they needed to be treated with any other antibiotic medication during the observation period.

### Clinical data acquisition

We gathered the following clinically and demographically relevant parameters: age, sex, weight, height, Charlson Comorbidity Score [26], laboratory parameters (creatinine, bilirubin, platelet count, neutrophils count, white blood cell count) at each sample collection time point, Glascow Coma Scale [27], and concurrent medication (virostatic agents, antifungals, anti-cancer drugs, proton pump inhibitors, cholesterol-lowering substances, and laxatives). A full list of administered concomitant medication is provided in Additional file 23: Table S13.

### Stool collection, DNA extraction, and shotgun metagenomic sequencing

In order to determine the intestinal resistome and to estimate the antibiotic-induced selection pressure, we collected four stool samples from each patient for shotgun metagenomics. The baseline sample T0 was collected within a maximum of 3 days before the start of antibiotic prophylaxis. Sample T1 was collected 1 day after initiation of prophylaxis, sample T2 after 3 days of prophylaxis, and sample T3 at the end of the observation period. The end of the observation period was either at the end of prophylactic drug administration or after 7 days of prophylaxis. A delay of up to + 48 h was tolerated for each time point. Between two time points, a minimum of 24 h must have been passed.

We collected stool samples in a sterile plastic device (Commode Specimen Collection System, Thermo Fisher Scientific, Pittsburgh, USA). The majority of stool samples were collected in the hospitals, while a few were collected at the patients’ home and immediately transported in cool bags to our laboratory. Samples were stored within 30 min at 4 °C and DNA was extracted on the same day using the Power Soil DNA Isolation Kit (Qiagen, Hilden, Germany). Shotgun metagenomic sequencing was carried out at the GATC Biotech AG (Konstanz, Germany) using the NEBNext Ultra DNA Library kit (New England Biolabs, Ipswich, USA) for DNA library preparation and an Illumina HiSeq platform for sequencing. A paired-end sequencing approach with a targeted read length of 150 bp and an insert size of 550 bp was conducted.

### Metagenomic assembly

Trimmomatic (version 0.35) was used to acquire high-quality reads [28]. Quality control of trimmed reads was performed with FastQC version 0.11.5 (https://www.bioinformatics.babraham.ac.uk/projects/fastqc/). We used SPAdes (version 3.9.0) to assemble metagenomic scaffolds with a minimum length of 1000 bp [29].

### Taxonomic classification, resistome identification, and definitions

Human contamination was removed by mapping reads against the human genome (GRCh38) using KneadData (https://bitbucket.org/biobakery/kneaddata/wiki/Home). Taxonomic profiling was carried out with Kaiju (version 1.5.0) using the greedy mode with a minimum alignment length of 11 amino acids, a maximum of 1 mismatch, and a match score of 65 [30]. The non-redundant protein database nr was used for classification. Counts for taxonomic units were normalized to a relative abundance through dividing the hits by the sample read count and multiplying the quotient by 10^6^. The resulting unit is hits per million reads (HPM).

In order to determine the resistome composition, we performed a blastx of decontaminated reads against the ARG-ANNOT database (AA, version 3) [13] using DIAMOND (version 0.8.0.62) [31]. We set the query cover to 75% and used the “sensitive” mode as well as a best hit algorithm where one read is only assigned to one database entry based on the best bit-score. Hits against antibiotic resistance genes (ARGs) were transformed into length-corrected relative abundance (LCRA). As with the taxonomic units, we calculated the HPM for each ARG. For LCRA calculation, we divided the HPM by the respective ARG length in kilobase and acquired the unit HPM per kilobase gene length. LCRAs for ARG classes were calculated by summing up the individual ARG LCRAs that belong to the respective ARG class.

### Baseline-endpoint comparison for the illustration of antibiotic impact

We performed a baseline-endpoint comparison (BEC) by subtracting the baseline value (antibiotic naïve patient, T0) from the value at T3 (end of observation period). BEC reflects an overall crude treatment effect. Positive values illustrate an increase of the respective factor, negative values a decrease.

### Determination and normalization of antimicrobial selection pressure

This second strategy to determine antimicrobial selection pressure has previously been reported in detail by our group and has been validated using qPCR [10]. Briefly, we account for individual heterogeneity within the time series data using fixed- or random-effects models. The coefficients of the models express a change in an investigated outcome value by a defined unit increase of the model components, as for instance the increase or decrease of ARG LCRA per defined daily dose (DDD) of an antibiotic or another drug. Of note, time series data from each patient were clustered within a model, thus providing more conservative standard errors. This way, each patient was considered his or her own control by comparing the baseline sample with the other time points. Finally, the regression models always report the overall effect for a cohort.

### Regression modeling normalization and multivariate regression procedure

Potential contributors were identified through analyzing which factors were significantly distinct in both treatment groups. A univariate analysis was performed investigating the relationship between antibiotic treatment and all identified potential contributors with each investigated outcome. If a model’s component was found to have a significant impact on the outcome (*p* ≤ 0.05), it was included into the final multivariate model. Antibiotic treatment as primary exposure of interest was always included as a component in the final multivariate model. This way, we were able to estimate the independent degree of selection pressure caused by antibiotics and other variables.

In order to improve comparison between coefficients with different units and data ranges, we normalized the regression model coefficients by dividing a coefficient with the population mean of the baseline samples. This quotient was subsequently multiplied by 100. Population means were calculated and applied for both treatment groups. The resulting unit is an average percentage increase/decrease of the observed outcome per unit of the model component. One example would be an average 148.1% increase in the abundance of sulfonamide resistance genes per administered DDD of cotrimoxazole.

A statistical comparison between the effects of both drugs was performed by including the coefficients for the same outcome and model component in a nested likelihood ratio test. A Bonferroni-corrected LR *p* value < 0.002 was regarded a significant difference in the impact of both antibiotics on a specific ARG class.

### Beta-lactamase antibiotic resistance gene investigation

OXA, TEM, and SHV hits were investigated differently. Metagenomic scaffolds were submitted to a blastx against ARG-ANNOT. ARGs on the scaffolds with 100% sequence similarity to database entries were documented, and only hits versus such ARGs were further investigated. TEM and SHV genes must have been members of the functional group 2be according to Bush-Jacoby [32]. ARGs and ARG classes were only submitted to further analysis if hits were detected in at least 10 samples.

### Diversity and evenness definition and calculation

We calculated Shannon diversity and Simpson’s evenness for the composition of the sample microbiome and resistome. Shannon’s diversity H′ was calculated according to the following formula where *Pi* represents the proportion of counts within a certain unit *i* in relation to the total population count. In this case, a unit count could be the HPM of a taxon or the LCRA of an ARG or ARG class.

$$ {H}^{\prime }=-\sum Pi\ \ln (Pi) $$

Simpson’s evenness *E* was calculated based on Simpson’s dominance *D*_2_.

$$ {D}_2=1/\sum {Pi}^2 $$

The evenness *E* was then determined by dividing *D*_2_ by the total number of individual units (richness). Here again, units could be taxa, ARGs, or ARG classes. We have used the same formulas for the calculation of plasmid diversity and evenness.

### Definition of species emergence and disappearance within the gut microbiome

The emergence and potential colonization of a species was defined as no detection of the species at baseline but detection at a minimum of two time points during treatment and detection at T3. A disappearance and potential decolonization of a species was defined as detection at baseline but no detection at T2 and T3. Counts of emerging and disappearing species were generated for each patient and compared to the total species count detected at baseline. The means of these values were assumed to reflect species emergence and disappearance events under both antibiotics.

### Analysis of ciprofloxacin-mediating mutations

The ARG-ANNOT database includes mostly plasmid-mediated fluoroquinolone resistance proteins (Qnr). However, fluoroquinolone resistance is also mediated by target modifications and overexpression of multi-drug efflux pumps [33]. Apart from QepA, NorA, OqxA, and OqxB, no other efflux pumps are included in ARG-ANNOT. For this reason, we looked specifically for these other mechanisms of resistance.

We investigated mutations reported to increase the MIC of fluoroquinolones in the following proteins: GyrA (S83L, D87N, D87T), ParC (S80I, E84V, E84G), ParE (S458A, E460D), AcrR (R45C), AcrB (G288D). We used the wild-type *Escherichia coli* strain K-12 MG1655 as reference. We also investigated potential mutations in Gram-positive organisms in the following proteins: GyrA (S84L with *Staphylococcus aureus* NCTC8225 as reference) and ParC (S80F and E84K with *Staphylococcus aureus* NCTC8325 as reference). SNPs at these positions were called by mapping the sequence reads against these references using BWA (version 0.6.2) and samtools (version 1.2) with a mapping and quality score of 30 [34, 35]. We counted the proportion of reads showing the mutation (dp4 values) and calculated the percentage difference between the baseline sample (antibiotic treatment naïve) and T3 (end of observation period). A relevant increase in the proportion of reads carrying fluoroquinolone resistance-mediating mutations was considered a positive selection under treatment.

### Gut plasmid content determination and definitions

The plasmidome was identified using PlasFlow (version 1.1) based on the scaffolds from our metagenomic assembly [36]. Based on a threshold of 0.7, PlasFlow categorized each scaffold according to its taxonomic ancestry and indicated it to be either of chromosomal or plasmid origin.

### Plasmid abundance and total plasmid-ARG content determination

For calculating plasmid abundance in one sample, we determined the sum of coverages for all identified plasmids and divided this sum by the sample read count. Subsequently, this quotient was multiplied by 10^6^, resulting in an expected coverage sum per million input reads (normalized coverage). For the sake of simplicity, we termed this normalized coverage sum the “plasmid abundance.” For calculating diversity and evenness, we normalized the coverage of each plasmid by dividing it by the sample read count and multiplying the quotient by 10^6^. This value was regarded as normalized coverage for an individual plasmid. Following the previous concept, we also normalized plasmid richness (number of unique plasmids) by dividing the richness by the sample read count and multiplying the quotient by 10^6^, acquiring a normalized richness.

Genes on plasmid scaffolds from each sample and taxonomic origin were predicted using Prokka (version 1.11) [37]. Predicted genes were clustered by CD-HIT-EST (version 4.6) [38] using the following options: -c 0.98 -aL 0.9 -aS 0.9. Subsequently, we performed a blastx (version 2.3.0) against the ARG-ANNOT database (max_target_seqs 25) [39]. For each sample, we determined the number of ARGs from each ARG class from plasmids of different taxonomic origin. The sum of ARG hits was considered the sample ARG class abundance from the plasmids of the respective origin. The total sum of ARG class abundances from all samples of a sampling time point was regarded as the total plasmid-ARG content, reflecting both ARG abundance as well as ARG richness on plasmids within the patient population. A co-occurrence network from each sampling time point was drawn based on this total plasmid-ARG content which is reflected by the line width.

### Correlation analyses and resistance score generation

All phyla and species were correlated with the most abundant ARG classes using Kendall’s rank correlation. We also built a correlation matrix between baseline taxonomic and plasmidome diversities, ARG class BEC values, and a resistance score using Spearman’s rank correlation. The resistance score was computed for each patient and was based on the BEC values of the 11 most abundant ARG classes. For each ARG class, a positive BEC was scored as one, a negative as zero. The points for all ARG classes were summed up and attributed to the respective patient. The score ranges between 0 and 11, with higher values indicating an overall stronger positive ARG selection.

### Statistical analysis

D’Agostino’s *K*-squared test was used to examine continuous variables for normality, Bartlett’s test for equality of variances. The chi-squared test was applied for hypothesis testing regarding observed frequencies on one or more categories. Differences of continuous parameter distributions were assessed with either Student’s *t* test or the Wilcoxon rank-sum test. A *p* value < 0.05 (two-sided) was considered statistically significant. Statistical analyses were conducted using either Stata version 12.1 (Stat Corp., College Station, TX, USA) or the Python-based Anaconda software suite (https://anaconda.org/).

## Additional files

Additional file 1: **Figure S1.** Flowchart of study participant recruitment and exclusion reasons. (PDF 196 kb)

Additional file 2: **Table S1.** Time period between hospital admission and baseline stool sampling for both cohorts and each patient. (PDF 37 kb)

Additional file 3: **Table S2.** Baseline disparities in the intestinal microbiome between BEC groups (PDF 48 kb)

Additional file 4: **Table S3.** Multivariate models of selection pressure estimates on the intestinal microbiome (PDF 45 kb)

Additional file 5: **Table S4.** Univariate models of selection pressure estimates on the intestinal microbiome (PDF 44 kb)

Additional file 6: **Figure S2.** Microbiome diversity and evenness trajectories. A heatmap with microbiome diversity trajectories at phylum level is displayed in (A), at species level in (B), of microbiome evenness at phylum level in (C), and at species level in (D). Microbiome diversity was calculated using the Shannon index, and evenness using the reciprocal of Simpson’s dominance (see patients and methods). Diversity and evenness values are printed in all boxes for each study participant (Patient ID, on y-axis) and day of stool collection (T0 - T3, on x-axis). Treatment period was from T1 to T3. T0 is the sample before antibiotic exposures. Orange heatmaps include patients from the ciprofloxacin cohort, blue heatmaps patients from the cotrimoxazole cohort. The bottom row of the heatmaps presents the mean value of each column, here the sample collection time point. The intensity of the color bar reflects the diversity and evenness values, with a deeper color for higher values. (PDF 1281 kb)

Additional file 7: **Figure S3.** Trajectories of richness, diversity and evenness of both cohorts over the entire observation period. Trajectories of richness, Shannon diversity and Simpson’s evenness before treatment (T0), at T1, at T2, and at the end of the observation period (T3) are shown on phylum rank (A) and species rank (B) for both antibiotic treatments. Blue data points are measurements at T0, yellow data points at T1, green data points at T2, and dark orange data points at T3. Boxplots indicate the distribution of data. The connecting magenta line shows the means at each time point and their development under treatment. Under ciprofloxacin treatment, richness and Shannon diversity decrease significantly while Simpson’s evenness remains stable. In contrast, under cotrimoxazole, loss of richness and diversity is less pronounced. (PDF 2405 kb)

Additional file 8: **Figure S4.** Comparison of length corrected relative abundances from antimicrobial resistance gene classes before treatment and at the end of observation. Trajectories of antimicrobial resistance genes quantification by LCRA before treatment (T0) and at the end of the observation period (T3) are shown for both antibiotic treatments. Pink data points are measurements at T0, purple data points at T3. Boxplots indicate the distribution of data. The connecting magenta line shows the means at each time point and their development under treatment (paired *t*-test). Trends for LCRA changes are prominent but do not reach statistical significance. The following ARG classes are depicted: aminoglycosides (AGly), beta-lactamases (Bla), fluoroquinolones (Flq), glycopeptides (Gly), macrolide-lincosamide-streptogramin (MLS), nitroimidazoles (Ntmdz), phenicols (Phe), sulfonamides (Sul), tetracyclines (tet), and trimethoprim (Tmt). CTX-M are cefotaximase enzymes that mediate an ESBL phenotype. (PDF 2247 kb)

Additional file 9: **Figure S5.** Abundance trajectories of aminoglycoside and fluoroquinolone antibiotic resistance gene classes und CTX-M as well as beta-lactamases. Abundance heatmaps of the aminoglycoside antibiotic resistance gene (ARG) class are displayed in (A), of beta-lactamases in (B), of CTX-M in (C), and of the fluoroquinolone ARG class in (D). Abundances are expressed as square root transformed length corrected relative abundances (LCRA, see patients and methods). LCRA values are printed in all boxes for each study participant (Patient ID, on y-axis) and day of stool collection (T0 - T3, on x-axis). Treatment period was from T1 to T3. T0 is the sample before antibiotic exposures. Orange heatmaps include patients from the ciprofloxacin cohort, blue heatmaps patients from the cotrimoxazole cohort. The bottom row of the heatmaps presents the mean value of each column, here the sample collection time point. The intensity of the color bar reflects the LCRA values, with a deeper color for higher values. (PDF 948 kb)

Additional file 10: **Figure S6.** Abundance trajectories of glycopeptide, macrolide-lincosamide-streptogramin, nitroimidazole and phenicol antibiotic resistance gene classes. Abundance heatmaps of the glycopeptide antibiotic resistance gene (ARG) class are displayed in (A), of the macrolide-lincosamide-streptogramin (MLS) ARG class in (B), of the nitroimidazole ARG class in (C), and of phenicol ARG class in (D). Abundances are expressed as square root transformed length corrected relative abundances (LCRA, see patients and methods). LCRA values are printed in all boxes for each study participant (Patient ID, on y-axis) and day of stool collection (T0 - T3, on x-axis). Treatment period was from T1 to T3. T0 is the sample before antibiotic exposures. Orange heatmaps include patients from the ciprofloxacin cohort, blue heatmaps patients from the cotrimoxazole cohort. The bottom row of the heatmaps presents the mean value of each column, here the sample collection time point. The intensity of the color bar reflects the LCRA values, with a deeper color for higher values. (PDF 1023 kb)

Additional file 11: **Figure S7.** Abundance trajectories of sulfonamides, tetracyclin and trimethoprim antibiotic resistance gene classes. Abundance heatmaps of the sulfonamide antibiotic resistance gene (ARG) class are displayed in (A), of the tetracycline ARG class in (B), and of the trimethoprim ARG class in (C). Abundances are expressed as square root transformed length corrected relative abundances (LCRA, see patients and methods). LCRA values are printed in all boxes for each study participant (Patient ID, on y-axis) and day of stool collection (T0 - T3, on x-axis). Treatment period was from T1 to T3. T0 is the sample before antibiotic exposures. Orange heatmaps include patients from the ciprofloxacin cohort, blue heatmaps patients from the cotrimoxazole cohort. The bottom row of the heatmaps presents the mean value of each column, here the sample collection time point. The intensity of the color bar reflects the LCRA values, with a deeper color for higher values. (PDF 727 kb)

Additional file 12: **Figure S8.** Trajectories of length-corrected relative abundances of diverse antimicrobial resistance gene classes of both cohorts over the entire observation period. Trajectories of antimicrobial resistance genes quantification by LCRA before treatment (T0), at T1, at T2, and at the end of the observation period (T3) are shown for both antibiotic treatments. Blue data points are measurements at T0, yellow data points at T1, green data points at T2, and dark orange data points at T3. Boxplots indicate the distribution of data. The connecting magenta line shows the means at each time point and their development under treatment. The following ARG classes are depicted: aminoglycosides (AGly), beta-lactamases (Bla), fluoroquinolones (Flq), glycopeptides (Gly), macrolide-lincosamide-streptogramin (MLS), nitroimidazoles (Ntmdz), phenicols (Phe), sulfonamides (Sul), tetracyclines (tet), and trimethoprim (Tmt). CTX-M are cefotaximase enzymes that mediate an ESBL phenotype. (PDF 3083 kb)

Additional file 13: **Table S5.** Multivariate models of selection pressure estimates on antibiotic resistance genes. (PDF 59 kb)

Additional file 14: **Table S6.** Univariate models of selection pressure estimates on antibiotic resistance genes. (PDF 57 kb)

Additional file 15: **Table S7.** Baseline disparities in the intestinal resistome between BEC groups. (PDF 53 kb)

Additional file 16: **Figure S9.** Correlation matrix between gut resistome fractions and taxonomic units. A Kendall’s rank correlation matrix between taxonomic units (phylum level) and ARG classes for the ciprofloxacin cohort (A) und the cotrimoxazole cohort (B). The following ARG classes are depicted: aminoglycosides (AGly), beta-lactamases (Bla), fluoroquinolones (Flq), glycopeptides (Gly), macrolide-lincosamide-streptogramin (MLS), nitroimidazoles (Ntmdz), phenicols (Phe), sulfonamides (Sul), tetracyclines (tet), and trimethoprim (Tmt). (PDF 1180 kb)

Additional file 17: **Table S8.** Kendall’s rank correlation between gut microbiome species and gut resistome. (PDF 53 kb)

Additional file 18: **Table S9.** Baseline disparities in the intestinal plasmidome between BEC groups. (PDF 47 kb)

Additional file 19: **Figure S10.** Trajectories of plasmid abundance, plasmid diversity and plasmid evenness of both cohorts over the entire observation period. Trajectories of plasmid abundance, plasmid diversity and plasmid evenness before treatment (T0), at T1, at T2, and at the end of the observation period (T3) are shown for both antibiotic treatments. Blue data points are measurements at T0, yellow data points at T1, green data points at T2, and dark orange data points at T3. Boxplots indicate the distribution of data. The connecting magenta line shows the means at each time point and their development under treatment. (PDF 1651 kb)

Additional file 20: **Table S10.** Multivariate models of selection pressure estimates on the intestinal plasmidome. (PDF 46 kb)

Additional file 21: **Table S11.** Univariate models of selection pressure estimates on the intestinal plasmidome. (PDF 43 kb)

Additional file 22: **Table S12.** Complete list of inclusion and exclusion criteria (PDF 36 kb)

Additional file 23: **Table S13.** List of concomitant drugs in both study cohorts (PDF 46 kb)

## Data Availability

All metagenomic sequences used in this work (after removal of human sequences) are publicly accessible from the European Nucleotide Archive with the study accession number PRJEB28058 [40].
