# Supplementary figures and images for: Distinct impact of antibiotics on the gut microbiome and resistome: a longitudinal multicenter cohort study
Source: BMC Biol. 2019 Sep 18;17:76. doi: 10.1186/s12915-019-0692-y (PMC6749691; doi:10.1186/s12915-019-0692-y)

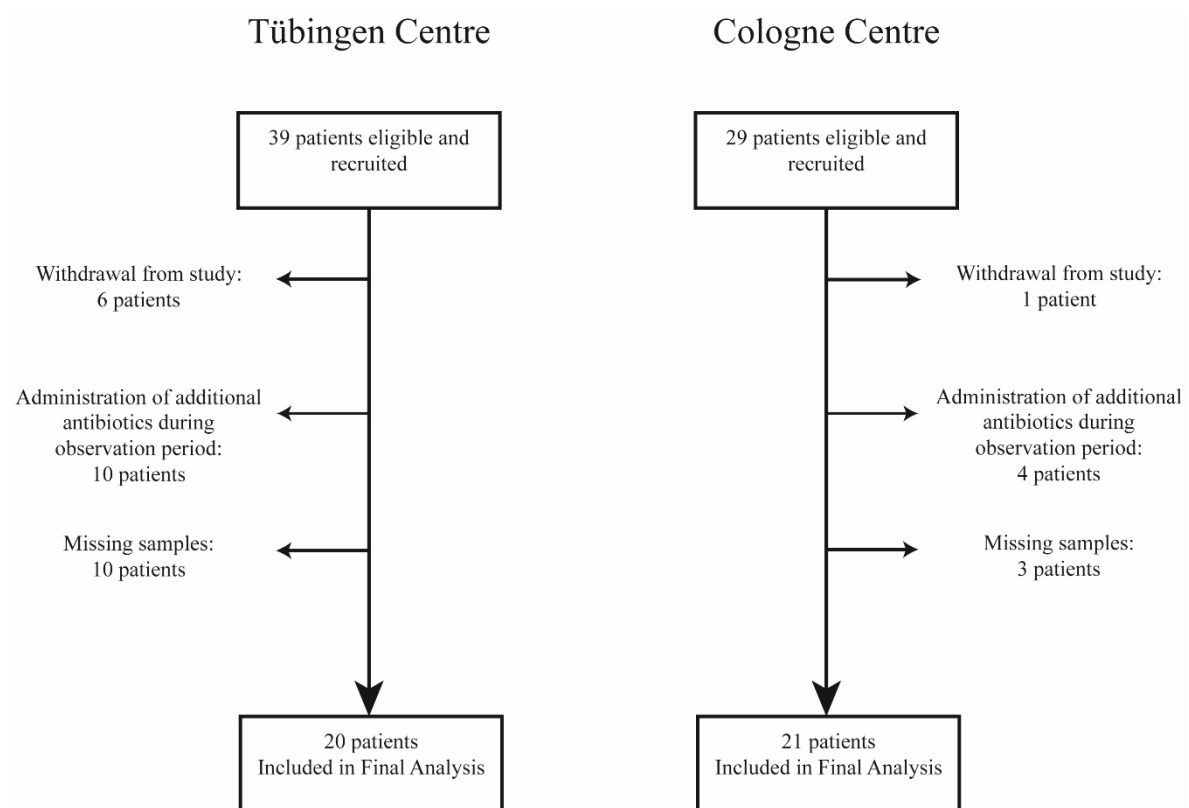

**Figure S1. Flowchart of study participant recruitment and exclusion reasons**

Supplement: Supplementary file 1 — Figure S1. Flowchart of study participant recruitment and exclusion reasons. (PDF 196 kb) [file 12915_2019_692_MOESM1_ESM.pdf]
