# Supplementary material for: Distinct impact of antibiotics on the gut microbiome and resistome: a longitudinal multicenter cohort study
Source: BMC Biol. 2019 Sep 18;17:76. doi: 10.1186/s12915-019-0692-y (PMC6749691; doi:10.1186/s12915-019-0692-y)
Supplement: Supplementary file 2 — Table S1. Time period between hospital admission and baseline stool sampling for both cohorts and each patient. (PDF 37 kb) [file 12915_2019_692_MOESM2_ESM.pdf]

Table S1. Time period between hospital admission and baseline stool sampling for both cohorts and each patient.

Cohort	ID	Hospital admission date	Date of baseline stool sample	Time to baseline stool (days)
Cologne (Cotrimoxazole)	1	26.01.2015	26.01.2015	0
	3	03.02.2015	04.02.2015	1
	4	19.02.2015	19.02.2015	0
	5	24.02.2015	25.02.2015	1
	7	28.04.2015	05.05.2015	7
	8	28.04.2015	29.04.2015	1
	10	28.05.2015	28.05.2015	0
	11	08.06.2015	08.06.2015	0
	12	22.06.2015	23.06.2015	1
	13	07.07.2016	08.07.2015	1
	17	10.09.2015	14.09.2015	4
	19	05.10.2015	06.10.2015	1
	20	05.11.2015	05.11.2015	0
	21	11.11.2015	12.11.2015	1
	23	07.12.2015	08.12.2015	1
	24	18.02.2016	21.02.2016	3
	25	03.05.2016	05.05.2016	2
	26	13.05.2016	17.05.2016	4
	27	18.05.2016	19.05.2016	1
	28	22.06.2016	23.06.2016	1
	29	23.06.2016	24.06.2016	1
	**Mean**			**1.47**
Tübingen (Ciprofloxacin)	504	01.10.2015	06.10.2015	6
	505	12.10.2015	13.10.2015	1
	506	14.10.2015	16.10.2015	2
	507	18.10.2015	20.10.2015	2
	508	26.10.2015	28.10.2015	2
	510	15.12.2015	18.12.2015	3
	511	02.02.2016	03.02.2016	1
	512	04.02.2016	09.02.2016	5
	516	22.03.2016	23.03.2016	1
	517	24.03.2016	25.03.2016	1
	518	04.05.2016	09.05.2016	5
	519	31.05.2016	01.06.2016	1
	522	13.06.2016	14.06.2016	1
	523	15.06.2016	16.06.2016	1
	524	28.06.2016	29.06.2016	1
	528	17.08.2016	18.08.2016	1
	532	20.09.2016	23.09.2016	3
	534	29.09.2016	29.09.2016	0
	537	19.10.2016	21.10.2016	2
	538	14.11.2016	14.11.2016	0
	**Mean**			**1.95**

ID, patient identifier.
