# Supplementary material for: Distinct impact of antibiotics on the gut microbiome and resistome: a longitudinal multicenter cohort study
Source: BMC Biol. 2019 Sep 18;17:76. doi: 10.1186/s12915-019-0692-y (PMC6749691; doi:10.1186/s12915-019-0692-y)
Supplement: Supplementary file 3 — Table S2. Baseline disparities in the intestinal microbiome between BEC groups (PDF 48 kb) [file 12915_2019_692_MOESM3_ESM.pdf]

Table S2. Baseline disparities in the intestinal microbiome between BEC groups

Microbiome parameter	Drug	Mean (Median), n Decrease Group	Mean (Median), n Increase Group	p-value
Phylum diversity	Ciprofloxacin	0.77 (0.77), 16	0.68 (0.65), 4	0.54
	Cotrimoxazole	0.86 (0.88), 15	0.67 (0.71), 6	0.05
Species diversity	Ciprofloxacin	4.51 (4.58), 20	n.a., 0	n.a.
	Cotrimoxazole	4.79 (4.89), 13	4.16 (4.06), 8	0.01
Phylum evenness	Ciprofloxacin	0.01 (0.01), 12	0.012 (0.011), 8	0.64
	Cotrimoxazole	0.012 (0.012), 15	0.01 (0.01), 6	0.06
Species evenness	Ciprofloxacin	0.0027 (0.0029), 12	0.0015 (0.0015), 8	0.006
	Cotrimoxazole	0.0023 (0.0024), 8	0.0016 (0.0016), 13	0.07

We have performed a comparison between data from the last time point and the baseline (T3 - T0, BEC). If a parameter did not change its BEC value or decreased (BEC value ≤ 0), the patient was part of the “Decrease Group”. If the BEC value was > 0, the patient was classified to belong to the “Increase Group”. Mean and median of baseline parameters from both groups are documented and hypothesis testing regarding a statistically significant difference was performed (p-value). ”n” denotes the respective group size.
