# Supplementary material for: Distinct impact of antibiotics on the gut microbiome and resistome: a longitudinal multicenter cohort study
Source: BMC Biol. 2019 Sep 18;17:76. doi: 10.1186/s12915-019-0692-y (PMC6749691; doi:10.1186/s12915-019-0692-y)
Supplement: Supplementary file 4 — Table S3. Multivariate models of selection pressure estimates on the intestinal microbiome (PDF 45 kb) [file 12915_2019_692_MOESM4_ESM.pdf]

Table S3. Multivariate models of selection pressure estimates on the intestinal microbiome

Microbiome variable	Drug model	Model components	Coefficient (95% CI)	p-value	LR p-value
Phylum Shannon diversity	Ciprofloxacin	CiproDDD	-0.03 (-0.05 - -0.01)	<0.001	0.62
		AF	0.13 (0.04 - 0.22)	0.004	
	Cotrimoxazole	CotrimDDD	-0.06 (-0.1 - -0.01)	0.012	
		Creatinin	0.2 (0.006 - 0.04)	0.008	
		VirosDDD	0.11 (0.08 - 0.15)	<0.001	
Species Shannon diversity	Ciprofloxacin	CiproDDD	-0.09 (-0.13 - -0.05)	<0.001	0.89
	Cotrimoxazole	CotrimDDD	-0.06 (-0.29 - 0.17)	0.62	
		Lymphoma	-0.76 (-0.92 - -0.6)	<0.001	
		Creatinin	0.1 (0.05 - 0.15)	<0.001	
		VirosDDD	-0.38 (-0.51 - -0.25)	<0.001	
		AFDDD	0.04 (0.006 - 0.08)	0.02	
Phylum evenness	Ciprofloxacin	CiproDDD	-0.00001 (-0.0002 - 0.0001)	0.86	0.18
		VirosDDD	0.004 (0.004 - 0.005)	<0.001	
	Cotrimoxazole	CotrimDDD	-0.0007 (-0.001 - -0.0001)	0.014	
		Creatinin	0.0002 (0.0001 - 0.0003)	0.001	
		VirosDDD	0.002 (0.0014 - 0.0021)	<0.0001	
Species evenness	Ciprofloxacin	CiproDDD	-0.00003 (-0.0001 - 0.00005)	0.51	0.8
	Cotrimoxazole	CotrimDDD	0.00005 (-0.0003 - 0.0004)	0.77	
		Lymphoma	-0.0008 (-0.002 - -0.00005)	0.04	
		Creatinin	0.0001 (0.00009 - 0.0002)	<0.001	

95% CI, 95% confidence interval; LR, likelihood ratio test for coefficient differences; CiproDDD, cumulative dose of ciprofloxacin in defined daily doses (DDD); CotrimDDD, cumulative dose of cotrimoxazole in defined daily doses (DDD); VirosDDD, cumulative dose of antiviral agents in defined daily doses (DDD); Lymphoma, lymphoma as underlying disease; AF, at least one administration of antifungals during the observation period, AFDDD, cumulative dose of antifungals in defined daily doses (DDD).

The coefficients denote the increase (positive coefficient) or decrease (negative coefficient) of the respective diversity/evenness per unit of the model component. For instance, a coefficient of -0.06 for CotrimDDD within phylum diversity means a decrease of 0.06 units Shannon diversity per cumulative DDD increase of cotrimoxazole. The p-value denotes the statistical significance of the coefficient. Contributing factors are displayed when statistical significant in the multivariate model ($p \leq 0.05$). The LR p-value indicates differences between the antibiotics' coefficients. A Bonferroni corrected LR p-value < 0.002 was regarded a significant difference in the impact of both antibiotics on a specific microbiome variable. The multivariate coefficient can be identical with the univariate coefficient for antibiotics when no confounding was noted.
