# Supplementary material for: Distinct impact of antibiotics on the gut microbiome and resistome: a longitudinal multicenter cohort study
Source: BMC Biol. 2019 Sep 18;17:76. doi: 10.1186/s12915-019-0692-y (PMC6749691; doi:10.1186/s12915-019-0692-y)
Supplement: Supplementary file 5 — Table S4. Univariate models of selection pressure estimates on the intestinal microbiome (PDF 44 kb) [file 12915_2019_692_MOESM5_ESM.pdf]

Table S4. Univariate models of selection pressure estimates on the intestinal microbiome

Microbiome variable	Drug model	Model components	Coefficient (95% CI)	p-value
Phylum Shannon diversity	Ciprofloxacin	CiproDDD	-0.03 (-0.04 - -0.01)	<0.001
		AF	0.13 (0.04 - 0.22)	0.004
	Cotrimoxazole	CotrimDDD	-0.05 (-0.08 - -0.009)	0.014
		Creatinin	0.02 (0.004 - 0.04)	0.014
		VirosDDD	-0.02 (-0.04 - -0.002)	0.03
Species Shannon diversity	Ciprofloxacin	CiproDDD	-0.09 (-0.13 - -0.05)	<0.001
	Cotrimoxazole	CotrimDDD	-0.03 (-0.2 - 0.14)	0.71
		Leucaemia	0.58 (0.14 - 1.02)	0.01
		Lymphoma	-0.78 (-1.05 - -0.51)	<0.001
		Creatinin	0.1 (0.04 - 0.15)	0.001
		VirosDDD	-0.21 (-0.28 - -0.14)	<0.001
		AFDDD	0.05 (0.01 - 0.08)	0.004
Phylum evenness	Ciprofloxacin	CiproDDD	-0.00005 (-0.0002 - 0.00009)	0.48
		Bilirubin	-0.001 (-0.003 - - 0.00006)	0.04
		VirosDDD	0.004 (0.004 - 0.005)	<0.001
	Cotrimoxazole	CotrimDDD	-0.0006 (-0.001 - -0.0001)	0.009
		Creatinin	0.0002 (0.00007 - 0.0003)	0.002
		VirosDDD	0.0002 (0.00002 - 0.0004)	0.03
Species evenness	Ciprofloxacin	CiproDDD	-0.00003 (-0.0001 - 0.00005)	0.51
	Cotrimoxazole	CotrimDDD	0.00007 (-0.0002 - 0.0003)	0.63
		Lymphoma	-0.0009 (-0.001 - -0.0003)	0.004
		Creatinin	0.0001 (0.00007 - 0.0002)	<0.001

95% CI, 95% confidence interval; LR, likelihood ratio test for coefficient differences; CiproDDD, cumulative dose of ciprofloxacin in defined daily doses (DDD); CotrimDDD, cumulative dose of cotrimoxazole in defined daily doses (DDD); VirosDDD, cumulative dose of antiviral agents in defined daily doses (DDD); Lymphoma, lymphoma as underlying disease; Leucaemia, leucaemia as underlying disease; AF, at least one administration of antifungals during the observation period, AFDDD, cumulative dose of antifungals in defined daily doses (DDD).

The coefficients denote the increase (positive coefficient) or decrease (negative coefficient) of the respective diversity/evenness per unit of the model component. For instance, a coefficient of -0.05 for CotrimDDD within phylum diversity means a decrease of 0.05 units Shannon diversity per cumulative DDD increase of cotrimoxazole. The p-value denotes the statistical significance of the coefficient. Contributing factors significant in the univariate model are displayed.
