# Supplementary material for: Distinct impact of antibiotics on the gut microbiome and resistome: a longitudinal multicenter cohort study
Source: BMC Biol. 2019 Sep 18;17:76. doi: 10.1186/s12915-019-0692-y (PMC6749691; doi:10.1186/s12915-019-0692-y)
Supplement: Supplementary file 6 — Figure S2. Microbiome diversity and evenness trajectories. A heatmap with microbiome diversity trajectories at phylum level is displayed in (A), at species level in (B), of microbiome evenness at phylum level in (C), and at species level in (D). Microbiome diversity was calculated using the Shannon index, and evenness using the reciprocal of Simpson’s dominance (see patients and methods). Diversity and evenness values are printed in all boxes for each study participant (Patient ID, on y-axis) and day of stool collection (T0 - T3, on x-axis). Treatment period was from T1 to T3. T0 is the sample before antibiotic exposures. Orange heatmaps include patients from the ciprofloxacin cohort, blue heatmaps patients from the cotrimoxazole cohort. The bottom row of the heatmaps presents the mean value of each column, here the sample collection time point. The intensity of the color bar reflects the diversity and evenness values, with a deeper color for higher values. (PDF 1281 kb) [file 12915_2019_692_MOESM6_ESM.pdf]

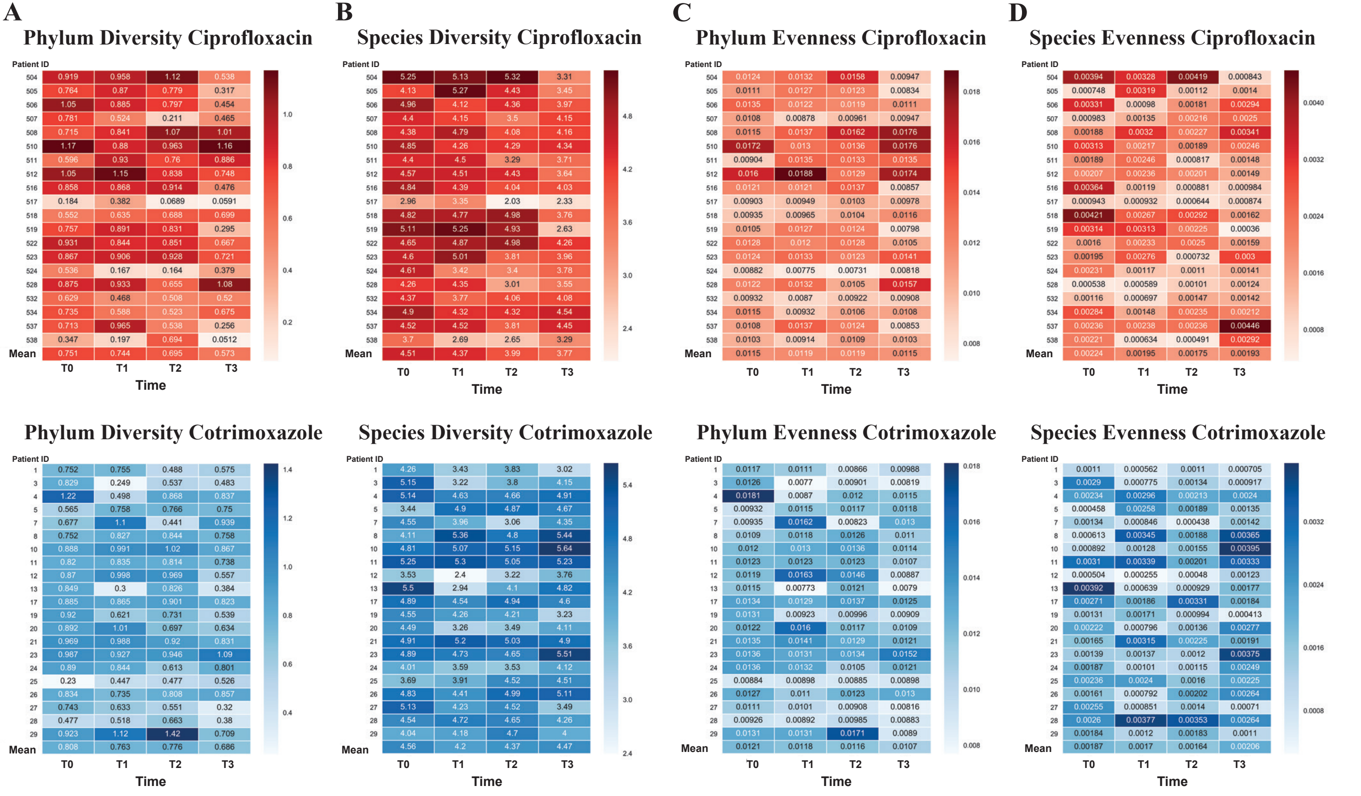
A
Phylum Diversity Ciprofloxacin
Patient ID
504 0.919 0.958 1.12 0.538
505 0.764 0.87 0.779 0.317
506 1.05 0.885 0.797 0.454
507 0.781 0.524 0.211 0.465
508 0.715 0.841 1.07 1.01
510 1.17 0.88 0.963 1.16
511 0.596 0.93 0.76 0.886
512 1.05 1.15 0.838 0.748
516 0.858 0.868 0.914 0.476
517 0.184 0.382 0.0689 0.0591
518 0.552 0.635 0.688 0.699
519 0.757 0.891 0.831 0.295
522 0.931 0.844 0.851 0.667
523 0.867 0.906 0.928 0.721
524 0.536 0.167 0.164 0.379
528 0.875 0.933 0.655 1.08
532 0.629 0.468 0.508 0.52
534 0.735 0.588 0.523 0.675
537 0.713 0.965 0.538 0.256
538 0.347 0.197 0.694 0.0512
Mean 0.751 0.744 0.695 0.573
T0 T1 T2 T3
Time
1.0 0.8 0.6 0.4 0.2
B
Species Diversity Ciprofloxacin
Patient ID
504 5.25 5.13 5.32 3.31
505 4.13 5.27 4.43 3.45
506 4.96 4.12 4.36 3.97
507 4.4 4.15 3.5 4.15
508 4.38 4.79 4.08 4.16
510 4.85 4.26 4.29 4.34
511 4.4 4.5 3.29 3.71
512 4.57 4.51 4.43 3.64
516 4.84 4.39 4.04 4.03
517 2.96 3.35 2.03 2.33
518 4.82 4.77 4.98 3.76
519 5.11 5.25 4.93 2.63
522 4.65 4.87 4.98 4.26
523 4.6 5.01 3.81 3.96
524 4.61 3.42 3.4 3.78
528 4.26 4.35 3.01 3.55
532 4.37 3.77 4.06 4.08
534 4.9 4.32 4.32 4.54
537 4.52 4.52 3.81 4.45
538 3.7 2.69 2.65 3.29
Mean 4.51 4.37 3.99 3.77
T0 T1 T2 T3
Time
4.8 4.2 3.6 3.0 2.4
C
Phylum Evenness Ciprofloxacin
Patient ID
504 0.0124 0.0132 0.0158 0.00947
505 0.0111 0.0127 0.0123 0.00834
506 0.0135 0.0122 0.0119 0.0111
507 0.0108 0.00878 0.00961 0.00947
508 0.0115 0.0137 0.0162 0.0176
510 0.0172 0.013 0.0136 0.0176
511 0.00904 0.0135 0.0133 0.0135
512 0.016 0.0188 0.0129 0.0174
516 0.0121 0.0121 0.0137 0.00857
517 0.00903 0.00949 0.0103 0.00978
518 0.00935 0.00965 0.0104 0.0116
519 0.0105 0.0127 0.0124 0.00798
522 0.0128 0.012 0.0128 0.0105
523 0.0124 0.0133 0.0123 0.0141
524 0.00882 0.00775 0.00731 0.00818
528 0.0122 0.0132 0.0105 0.0157
532 0.00932 0.0087 0.00922 0.00908
534 0.0115 0.00932 0.0106 0.0108
537 0.0108 0.0137 0.0124 0.00853
538 0.0103 0.00914 0.0109 0.0103
Mean 0.0115 0.0119 0.0119 0.0115
T0 T1 T2 T3
Time
0.018 0.016 0.014 0.012 0.010 0.008
D
Species Evenness Ciprofloxacin
Patient ID
504 0.00394 0.00328 0.00419 0.000843
505 0.000748 0.00319 0.00112 0.0014
506 0.00331 0.00098 0.00181 0.00294
507 0.000983 0.00135 0.00216 0.0025
508 0.00188 0.0032 0.00227 0.00341
510 0.00313 0.00217 0.00189 0.00246
511 0.00189 0.00246 0.000817 0.00148
512 0.00207 0.00236 0.00201 0.00149
516 0.00364 0.00119 0.000881 0.000984
517 0.000943 0.000932 0.000644 0.000874
518 0.00421 0.00267 0.00292 0.00162
519 0.00314 0.00313 0.00225 0.00036
522 0.0016 0.00233 0.0025 0.00159
523 0.00195 0.00276 0.000732 0.003
524 0.00231 0.00117 0.0011 0.00141
528 0.000538 0.000589 0.00101 0.00124
532 0.00116 0.000697 0.00147 0.00142
534 0.00284 0.00148 0.00235 0.00212
537 0.00236 0.00238 0.00236 0.00446
538 0.00221 0.000634 0.000491 0.00292
Mean 0.00224 0.00195 0.00175 0.00193
T0 T1 T2 T3
Time
0.0040 0.0032 0.0024 0.0016 0.0008
Phylum Diversity Cotrimoxazole
Patient ID
1 0.752 0.755 0.488 0.575
3 0.829 0.249 0.537 0.483
4 1.22 0.498 0.868 0.837
5 0.565 0.758 0.766 0.75
7 0.677 1.1 0.441 0.939
8 0.752 0.827 0.844 0.758
10 0.888 0.991 1.02 0.867
11 0.82 0.835 0.814 0.738
12 0.87 0.998 0.969 0.557
13 0.849 0.3 0.826 0.384
17 0.885 0.865 0.901 0.823
19 0.92 0.621 0.731 0.539
20 0.892 1.01 0.697 0.634
21 0.969 0.988 0.92 0.831
23 0.987 0.927 0.946 1.09
24 0.89 0.844 0.613 0.801
25 0.23 0.447 0.477 0.526
26 0.834 0.735 0.808 0.857
27 0.743 0.633 0.551 0.32
28 0.477 0.518 0.663 0.38
29 0.923 1.12 1.42 0.709
Mean 0.808 0.763 0.776 0.686
T0 T1 T2 T3
Time
1.4 1.2 1.0 0.8 0.6 0.4
Species Diversity Cotrimoxazole
Patient ID
1 4.26 3.43 3.83 3.02
3 5.15 3.22 3.8 4.15
4 5.14 4.63 4.66 4.91
5 3.44 4.9 4.87 4.67
7 4.55 3.96 3.06 4.35
8 4.11 5.36 4.8 5.44
10 4.81 5.07 5.15 5.64
11 5.25 5.3 5.05 5.23
12 3.53 2.4 3.22 3.76
13 5.5 2.94 4.1 4.82
17 4.89 4.54 4.94 4.6
19 4.55 4.26 4.21 3.23
20 4.49 3.26 3.49 4.11
21 4.91 5.2 5.03 4.9
23 4.89 4.73 4.65 5.51
24 4.01 3.59 3.53 4.12
25 3.69 3.91 4.52 4.51
26 4.83 4.41 4.99 5.11
27 5.13 4.23 4.52 3.49
28 4.54 4.72 4.65 4.26
29 4.04 4.18 4.7 4
Mean 4.56 4.2 4.37 4.47
T0 T1 T2 T3
Time
5.4 4.8 4.2 3.6 3.0 2.4
Phylum Evenness Cotrimoxazole
Patient ID
1 0.0117 0.0111 0.00866 0.00988
3 0.0126 0.0077 0.00901 0.00819
4 0.0181 0.0087 0.012 0.0115
5 0.00932 0.0115 0.0117 0.0118
7 0.00935 0.0162 0.00823 0.013
8 0.0109 0.0118 0.0126 0.011
10 0.012 0.013 0.0136 0.0114
11 0.0123 0.0123 0.0123 0.0107
12 0.0119 0.0163 0.0146 0.00887
13 0.0115 0.00773 0.0121 0.0079
17 0.0134 0.0129 0.0137 0.0125
19 0.0131 0.00923 0.00996 0.00909
20 0.0122 0.016 0.0117 0.0109
21 0.0135 0.0141 0.0129 0.0121
23 0.0136 0.0131 0.0134 0.0152
24 0.0136 0.0132 0.0105 0.0121
25 0.00884 0.00898 0.00885 0.00898
26 0.0127 0.011 0.0123 0.013
27 0.0111 0.0101 0.00908 0.00816
28 0.00926 0.00892 0.00985 0.00883
29 0.0131 0.0131 0.0171 0.0089
Mean 0.0121 0.0118 0.0116 0.0107
T0 T1 T2 T3
Time
0.018 0.016 0.014 0.012 0.010 0.008
Species Evenness Cotrimoxazole
Patient ID
1 0.0011 0.000562 0.0011 0.000705
3 0.0029 0.000775 0.00134 0.000917
4 0.00234 0.00296 0.00213 0.0024
5 0.000458 0.00258 0.00189 0.00135
7 0.00134 0.000846 0.000438 0.00142
8 0.000613 0.00345 0.00188 0.00365
10 0.000892 0.00128 0.00155 0.00395
11 0.0031 0.00339 0.00201 0.00333
12 0.000504 0.000255 0.00048 0.00123
13 0.00392 0.000639 0.000929 0.00177
17 0.00271 0.00186 0.00331 0.00184
19 0.00131 0.00171 0.000994 0.000413
20 0.00222 0.000796 0.00136 0.00277
21 0.00165 0.00315 0.00225 0.00191
23 0.00139 0.00137 0.0012 0.00375
24 0.00187 0.00101 0.00115 0.00249
25 0.00236 0.0024 0.0016 0.00225
26 0.00161 0.000792 0.00202 0.00264
27 0.00255 0.000851 0.0014 0.00071
28 0.0026 0.00377 0.00353 0.00264
29 0.00184 0.0012 0.00183 0.0011
Mean 0.00187 0.0017 0.00164 0.00206
T0 T1 T2 T3
Time
0.0032 0.0024 0.0016 0.0008

Figure S2. Microbiome diversity and evenness trajectories

A heatmap with microbiome diversity trajectories at phylum level is displayed in (A), at species level in (B), of microbiome evenness at phylum level in (C), and at species level in (D). Microbiome diversity was calculated using the Shannon index, and evenness using the reciprocal of Simpson's dominance (see patients and methods). Diversity and evenness values are printed in all boxes for each study participant (Patient ID, on y-axis) and day of stool collection (T0 - T3, on x-axis). Treatment period was from T1 to T3. T0 is the sample before antibiotic exposures. Orange heatmaps include patients from the ciprofloxacin cohort, blue heatmaps patients from the cotrimoxazole cohort. The bottom row of the heatmaps presents the mean value of each column, here the sample collection time point. The intensity of the color bar reflects the diversity and evenness values, with a deeper color for higher values.
