# Supplementary material for: Distinct impact of antibiotics on the gut microbiome and resistome: a longitudinal multicenter cohort study
Source: BMC Biol. 2019 Sep 18;17:76. doi: 10.1186/s12915-019-0692-y (PMC6749691; doi:10.1186/s12915-019-0692-y)
Supplement: Supplementary file 7 — Figure S3. Trajectories of richness, diversity and evenness of both cohorts over the entire observation period. Trajectories of richness, Shannon diversity and Simpson’s evenness before treatment (T0), at T1, at T2, and at the end of the observation period (T3) are shown on phylum rank (A) and species rank (B) for both antibiotic treatments. Blue data points are measurements at T0, yellow data points at T1, green data points at T2, and dark orange data points at T3. Boxplots indicate the distribution of data. The connecting magenta line shows the means at each time point and their development under treatment. Under ciprofloxacin treatment, richness and Shannon diversity decrease significantly while Simpson’s evenness remains stable. In contrast, under cotrimoxazole, loss of richness and diversity is less pronounced. (PDF 2405 kb) [file 12915_2019_692_MOESM7_ESM.pdf]

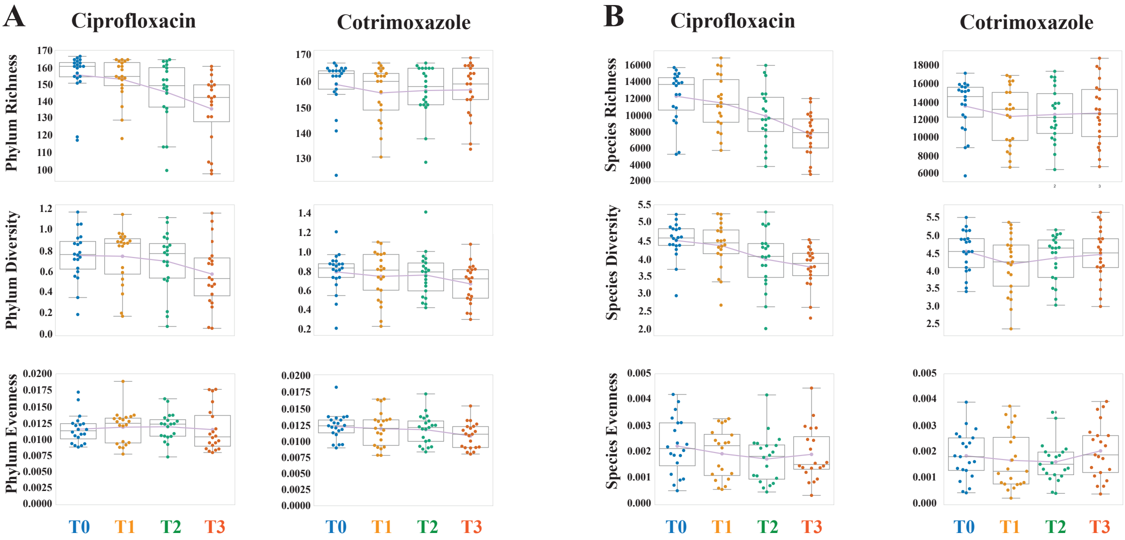
A
Ciprofloxacin
Cotrimoxazole
B
Ciprofloxacin
Cotrimoxazole
Phylum Richness
Phylum Diversity
Phylum Evenness
Species Richness
Species Diversity
Species Evenness
T0
T1
T2
T3

Figure S3. Trajectories of richness, diversity and evenness of both cohorts over the entire observation period.

Trajectories of richness, Shannon diversity and Simpson's evenness before treatment (T0), at T1, at T2, and at the end of the observation period (T3) are shown on phylum rank **(A)** and species rank **(B)** for both antibiotic treatments. Blue data points are measurements at T0, yellow data points at T1, green data points at T2, and dark orange data points at T3. Boxplots indicate the distribution of data. The connecting magenta line shows the means at each time point and their development under treatment. Under ciprofloxacin treatment, richness and Shannon diversity decrease significantly while Simpson's evenness remains stable. In contrast, under cotrimoxazole, loss of richness and diversity is less pronounced.
