# Supplementary material for: Distinct impact of antibiotics on the gut microbiome and resistome: a longitudinal multicenter cohort study
Source: BMC Biol. 2019 Sep 18;17:76. doi: 10.1186/s12915-019-0692-y (PMC6749691; doi:10.1186/s12915-019-0692-y)
Supplement: Supplementary file 8 — Figure S4. Comparison of length corrected relative abundances from antimicrobial resistance gene classes before treatment and at the end of observation. Trajectories of antimicrobial resistance genes quantification by LCRA before treatment (T0) and at the end of the observation period (T3) are shown for both antibiotic treatments. Pink data points are measurements at T0, purple data points at T3. Boxplots indicate the distribution of data. The connecting magenta line shows the means at each time point and their development under treatment (paired t-test). Trends for LCRA changes are prominent but do not reach statistical significance. The following ARG classes are depicted: aminoglycosides (AGly), beta-lactamases (Bla), fluoroquinolones (Flq), glycopeptides (Gly), macrolide-lincosamide-streptogramin (MLS), nitroimidazoles (Ntmdz), phenicols (Phe), sulfonamides (Sul), tetracyclines (tet), and trimethoprim (Tmt). CTX-M are cefotaximase enzymes that mediate an ESBL phenotype. (PDF 2247 kb) [file 12915_2019_692_MOESM8_ESM.pdf]

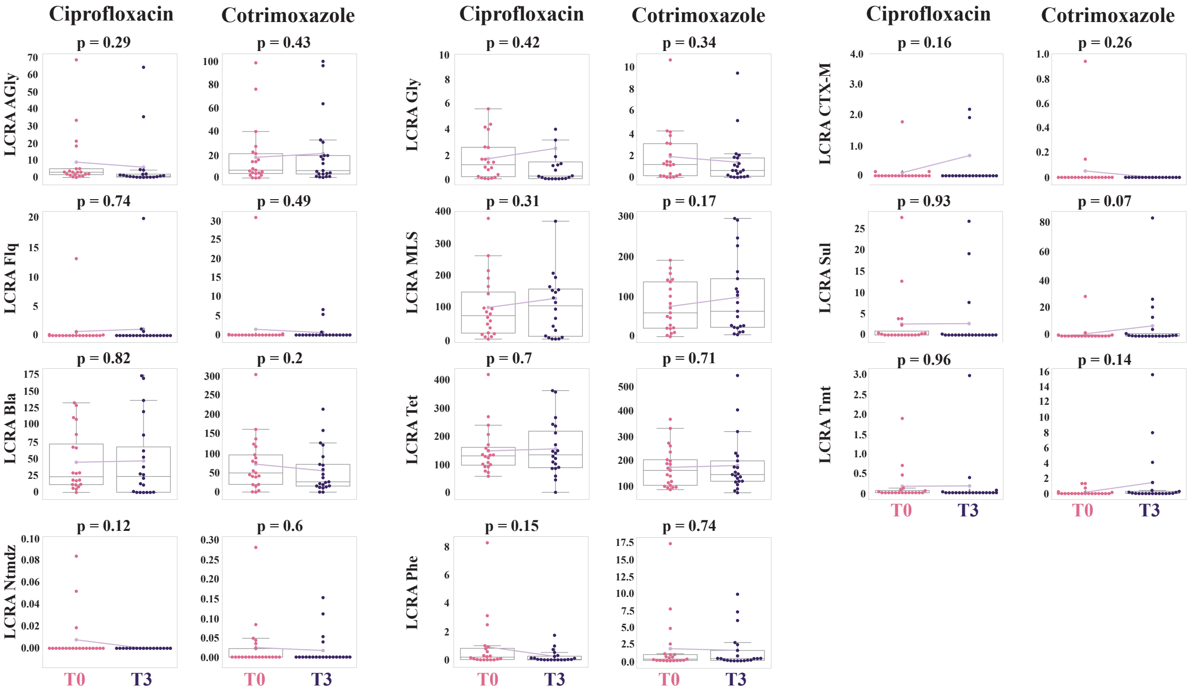
Ciprofloxacin
Cotrimoxazole
Ciprofloxacin
Cotrimoxazole
Ciprofloxacin
Cotrimoxazole
LCRA AGly
p = 0.29
p = 0.43
LCRA Gly
p = 0.42
p = 0.34
LCRA CTX-M
p = 0.16
p = 0.26
LCRA Flq
p = 0.74
p = 0.49
LCRA MLS
p = 0.31
p = 0.17
LCRA Sul
p = 0.93
p = 0.07
LCRA Bla
p = 0.82
p = 0.2
LCRA Tet
p = 0.7
p = 0.71
LCRA Tmt
p = 0.96
p = 0.14
LCRA Ntmdz
p = 0.12
p = 0.6
LCRA Phe
p = 0.15
p = 0.74
T0
T3

Figure S4. Comparison of length corrected relative abundances from antimicrobial resistance gene classes before treatment and at the end of observation.

Trajectories of antimicrobial resistance genes quantification by LCRA before treatment (T0) and at the end of the observation period (T3) are shown for both antibiotic treatments. Pink data points are measurements at T0, purple data points at T3. Boxplots indicate the distribution of data. The connecting magenta line shows the means at each time point and their development under treatment (paired t-test). Trends for LCRA changes are prominent but do not reach statistical significance. The following ARG classes are depicted: aminoglycosides (AGly), beta-lactamases (Bla), fluoroquinolones (Flq), glycopeptides (Gly), macrolide-lincosamide-streptogramin (MLS), nitroimidazoles (Ntmdz), phenicols (Phe), sulfonamides (Sul), tetracyclines (tet), and trimethoprim (Tmt). CTX-M are cefotaximase enzymes that mediate an ESBL phenotype.
