# Supplementary material for: Distinct impact of antibiotics on the gut microbiome and resistome: a longitudinal multicenter cohort study
Source: BMC Biol. 2019 Sep 18;17:76. doi: 10.1186/s12915-019-0692-y (PMC6749691; doi:10.1186/s12915-019-0692-y)
Supplement: Supplementary file 9 — Figure S5. Abundance trajectories of aminoglycoside and fluoroquinolone antibiotic resistance gene classes und CTX-M as well as beta-lactamases. Abundance heatmaps of the aminoglycoside antibiotic resistance gene (ARG) class are displayed in (A), of beta-lactamases in (B), of CTX-M in (C), and of the fluoroquinolone ARG class in (D). Abundances are expressed as square root transformed length corrected relative abundances (LCRA, see patients and methods). LCRA values are printed in all boxes for each study participant (Patient ID, on y-axis) and day of stool collection (T0 - T3, on x-axis). Treatment period was from T1 to T3. T0 is the sample before antibiotic exposures. Orange heatmaps include patients from the ciprofloxacin cohort, blue heatmaps patients from the cotrimoxazole cohort. The bottom row of the heatmaps presents the mean value of each column, here the sample collection time point. The intensity of the color bar reflects the LCRA values, with a deeper color for higher values. (PDF 948 kb) [file 12915_2019_692_MOESM9_ESM.pdf]

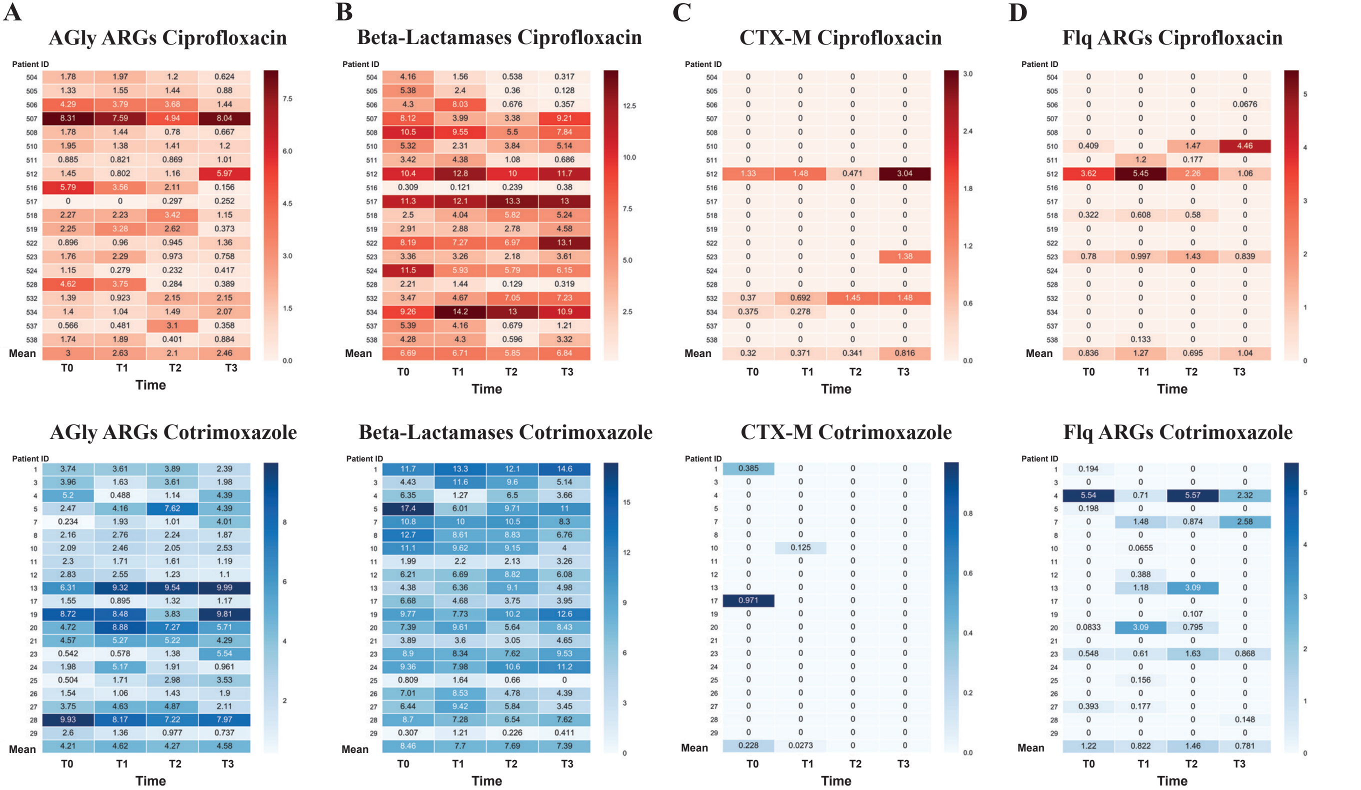
A
AGly ARGs Ciprofloxacin
B
Beta-Lactamases Ciprofloxacin
C
CTX-M Ciprofloxacin
D
Flq ARGs Ciprofloxacin
AGly ARGs Cotrimoxazole
Beta-Lactamases Cotrimoxazole
CTX-M Cotrimoxazole
Flq ARGs Cotrimoxazole
Patient ID
Mean
T0
T1
T2
T3
Time

Figure S5. Abundance trajectories of aminoglycoside and fluoroquinolone antibiotic resistance gene classes und CTX-M as well as beta-lactamases.

Abundance heatmaps of the aminoglycoside antibiotic resistance gene (ARG) class are displayed in (A), of beta-lactamases in (B), of CTX-M in (C), and of the fluoroquinolone ARG class in (D). Abundances are expressed as square root transformed length corrected relative abundances (LCRA, see patients and methods). LCRA values are printed in all boxes for each study participant (Patient ID, on y-axis) and day of stool collection (T0 - T3, on x-axis). Treatment period was from T1 to T3. T0 is the sample before antibiotic exposures. Orange heatmaps include patients from the ciprofloxacin cohort, blue heatmaps patients from the cotrimoxazole cohort. The bottom row of the heatmaps presents the mean value of each column, here the sample collection time point. The intensity of the color bar reflects the LCRA values, with a deeper color for higher values.
