# Supplementary material for: Distinct impact of antibiotics on the gut microbiome and resistome: a longitudinal multicenter cohort study
Source: BMC Biol. 2019 Sep 18;17:76. doi: 10.1186/s12915-019-0692-y (PMC6749691; doi:10.1186/s12915-019-0692-y)
Supplement: Supplementary file 10 — Figure S6. Abundance trajectories of glycopeptide, macrolide-lincosamide-streptogramin, nitroimidazole and phenicol antibiotic resistance gene classes. Abundance heatmaps of the glycopeptide antibiotic resistance gene (ARG) class are displayed in (A), of the macrolide-lincosamide-streptogramin (MLS) ARG class in (B), of the nitroimidazole ARG class in (C), and of phenicol ARG class in (D). Abundances are expressed as square root transformed length corrected relative abundances (LCRA, see patients and methods). LCRA values are printed in all boxes for each study participant (Patient ID, on y-axis) and day of stool collection (T0 - T3, on x-axis). Treatment period was from T1 to T3. T0 is the sample before antibiotic exposures. Orange heatmaps include patients from the ciprofloxacin cohort, blue heatmaps patients from the cotrimoxazole cohort. The bottom row of the heatmaps presents the mean value of each column, here the sample collection time point. The intensity of the color bar reflects the LCRA values, with a deeper color for higher values. (PDF 1023 kb) [file 12915_2019_692_MOESM10_ESM.pdf]

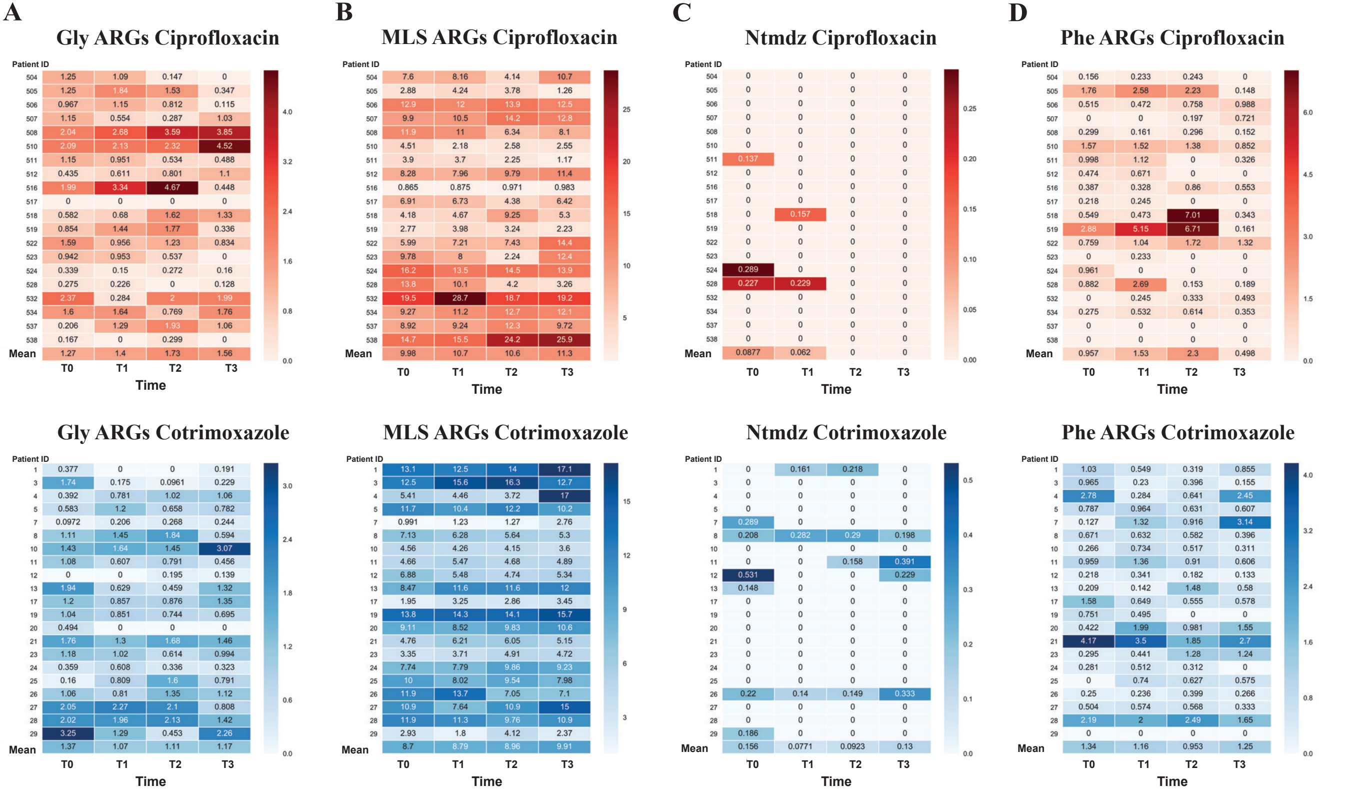
A
Gly ARGs Ciprofloxacin
B
MLS ARGs Ciprofloxacin
C
Ntmdz Ciprofloxacin
D
Phe ARGs Ciprofloxacin
Gly ARGs Cotrimoxazole
MLS ARGs Cotrimoxazole
Ntmdz Cotrimoxazole
Phe ARGs Cotrimoxazole
Patient ID
Mean
T0
T1
T2
T3
Time

**Figure S6. Abundance trajectories of glycopeptide, macrolide-lincosamide-streptogramin, nitroimidazole and phenicol antibiotic resistance gene classes.** Abundance heatmaps of the glycopeptide antibiotic resistance gene (ARG) class are displayed in (A), of the macrolide-lincosamide-streptogramin (MLS) ARG class in (B), of the nitroimidazole ARG class in (C), and of phenicol ARG class in (D). Abundances are expressed as square root transformed length corrected relative abundances (LCRA, see patients and methods). LCRA values are printed in all boxes for each study participant (Patient ID, on y-axis) and day of stool collection (T0 - T3, on x-axis). Treatment period was from T1 to T3. T0 is the sample before antibiotic exposures. Orange heatmaps include patients from the ciprofloxacin cohort, blue heatmaps patients from the cotrimoxazole cohort. The bottom row of the heatmaps presents the mean value of each column, here the sample collection time point. The intensity of the color bar reflects the LCRA values, with a deeper color for higher values.
