# Supplementary material for: Distinct impact of antibiotics on the gut microbiome and resistome: a longitudinal multicenter cohort study
Source: BMC Biol. 2019 Sep 18;17:76. doi: 10.1186/s12915-019-0692-y (PMC6749691; doi:10.1186/s12915-019-0692-y)
Supplement: Supplementary file 11 — Figure S7. Abundance trajectories of sulfonamides, tetracyclin and trimethoprim antibiotic resistance gene classes. Abundance heatmaps of the sulfonamide antibiotic resistance gene (ARG) class are displayed in (A), of the tetracycline ARG class in (B), and of the trimethoprim ARG class in (C). Abundances are expressed as square root transformed length corrected relative abundances (LCRA, see patients and methods). LCRA values are printed in all boxes for each study participant (Patient ID, on y-axis) and day of stool collection (T0 - T3, on x-axis). Treatment period was from T1 to T3. T0 is the sample before antibiotic exposures. Orange heatmaps include patients from the ciprofloxacin cohort, blue heatmaps patients from the cotrimoxazole cohort. The bottom row of the heatmaps presents the mean value of each column, here the sample collection time point. The intensity of the color bar reflects the LCRA values, with a deeper color for higher values. (PDF 727 kb) [file 12915_2019_692_MOESM11_ESM.pdf]

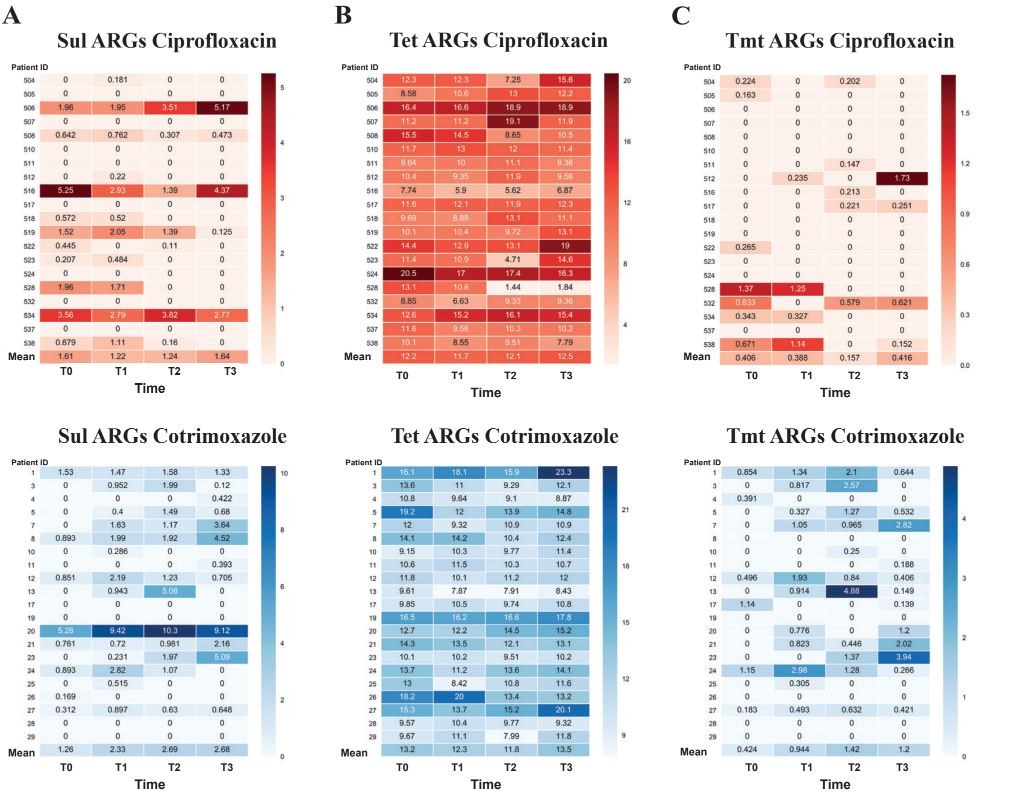
A
Sul ARGs Ciprofloxacin
Patient ID
504 0 0.181 0 0
505 0 0 0 0
506 1.96 1.95 3.51 5.17
507 0 0 0 0
508 0.642 0.762 0.307 0.473
510 0 0 0 0
511 0 0 0 0
512 0 0.22 0 0
516 5.25 2.93 1.39 4.37
517 0 0 0 0
518 0.572 0.52 0 0
519 1.52 2.05 1.39 0.125
522 0.445 0 0.11 0
523 0.207 0.484 0 0
524 0 0 0 0
528 1.96 1.71 0 0
532 0 0 0 0
534 3.56 2.79 3.82 2.77
537 0 0 0 0
538 0.679 1.11 0.16 0
Mean 1.61 1.22 1.24 1.64
T0 T1 T2 T3
Time
5 4 3 2 1 0
B
Tet ARGs Ciprofloxacin
Patient ID
504 12.3 12.3 7.25 15.6
505 8.58 10.6 13 12.2
506 16.4 16.6 18.9 18.9
507 11.2 11.2 19.1 11.9
508 15.5 14.5 8.65 10.5
510 11.7 13 12 11.4
511 9.64 10 11.1 9.36
512 10.4 9.35 11.9 9.56
516 7.74 5.9 5.62 6.87
517 11.6 12.1 11.9 12.3
518 9.69 8.88 13.1 11.1
519 10.1 10.4 9.72 13.1
522 14.4 12.9 13.1 19
523 11.4 10.9 4.71 14.6
524 20.5 17 17.4 16.3
528 13.1 10.8 1.44 1.84
532 8.85 6.63 9.33 9.36
534 12.6 15.2 16.1 15.4
537 11.6 9.58 10.3 10.2
538 10.1 8.55 9.51 7.79
Mean 12.2 11.7 12.1 12.5
T0 T1 T2 T3
Time
20 16 12 8 4
C
Tmt ARGs Ciprofloxacin
Patient ID
504 0.224 0 0.202 0
505 0.163 0 0 0
506 0 0 0 0
507 0 0 0 0
508 0 0 0 0
510 0 0 0 0
511 0 0 0.147 0
512 0 0.235 0 1.73
516 0 0 0.213 0
517 0 0 0.221 0.251
518 0 0 0 0
519 0 0 0 0
522 0.265 0 0 0
523 0 0 0 0
524 0 0 0 0
528 1.37 1.25 0 0
532 0.833 0 0.579 0.621
534 0.343 0.327 0 0
537 0 0 0 0
538 0.671 1.14 0 0.152
Mean 0.406 0.388 0.157 0.416
T0 T1 T2 T3
Time
1.5 1.2 0.9 0.6 0.3 0.0
Sul ARGs Cotrimoxazole
Patient ID
1 1.53 1.47 1.58 1.33
3 0 0.952 1.99 0.12
4 0 0 0 0.422
5 0 0.4 1.49 0.68
7 0 1.63 1.17 3.64
8 0.893 1.99 1.92 4.52
10 0 0.286 0 0
11 0 0 0 0.393
12 0.851 2.19 1.23 0.705
13 0 0.943 5.08 0
17 0 0 0 0
19 0 0 0 0
20 5.28 9.42 10.3 9.12
21 0.761 0.72 0.981 2.16
23 0 0.231 1.97 5.09
24 0.893 2.82 1.07 0
25 0 0.515 0 0
26 0.169 0 0 0
27 0.312 0.897 0.63 0.648
28 0 0 0 0
29 0 0 0 0
Mean 1.26 2.33 2.69 2.68
T0 T1 T2 T3
Time
10 8 6 4 2 0
Tet ARGs Cotrimoxazole
Patient ID
1 16.1 18.1 15.9 23.3
3 13.6 11 9.29 12.1
4 10.8 9.64 9.1 8.87
5 19.2 12 13.9 14.8
7 12 9.32 10.9 10.9
8 14.1 14.2 10.4 12.4
10 9.15 10.3 9.77 11.4
11 10.6 11.5 10.3 10.7
12 11.8 10.1 11.2 12
13 9.61 7.87 7.91 8.43
17 9.85 10.5 9.74 10.8
19 16.5 16.2 16.6 17.8
20 12.7 12.2 14.5 15.2
21 14.3 13.5 12.1 13.1
23 10.1 10.2 9.51 10.2
24 13.7 11.2 13.6 14.1
25 13 8.42 10.8 11.6
26 18.2 20 13.4 13.2
27 15.3 13.7 15.2 20.1
28 9.57 10.4 9.77 9.32
29 9.67 11.1 7.99 11.8
Mean 13.2 12.3 11.8 13.5
T0 T1 T2 T3
Time
21 18 15 12 9
Tmt ARGs Cotrimoxazole
Patient ID
1 0.854 1.34 2.1 0.644
3 0 0.817 2.57 0
4 0.391 0 0 0
5 0 0.327 1.27 0.532
7 0 1.05 0.965 2.82
8 0 0 0 0
10 0 0 0.25 0
11 0 0 0 0.188
12 0.496 1.93 0.84 0.406
13 0 0.914 4.88 0.149
17 1.14 0 0 0.139
19 0 0 0 0
20 0 0.776 0 1.2
21 0 0.823 0.446 2.02
23 0 0 1.37 3.94
24 1.15 2.98 1.28 0.266
25 0 0.305 0 0
26 0 0 0 0
27 0.183 0.493 0.632 0.421
28 0 0 0 0
29 0 0 0 0
Mean 0.424 0.944 1.42 1.2
T0 T1 T2 T3
Time
4 3 2 1 0

Figure S7. Abundance trajectories of sulfonamides, tetracyclin and trimethoprim antibiotic resistance gene classes.

Abundance heatmaps of the sulfonamide antibiotic resistance gene (ARG) class are displayed in (A), of the tetracycline ARG class in (B), and of the trimethoprim ARG class in (C). Abundances are expressed as square root transformed length corrected relative abundances (LCRA, see patients and methods). LCRA values are printed in all boxes for each study participant (Patient ID, on y-axis) and day of stool collection (T0 - T3, on x-axis). Treatment period was from T1 to T3. T0 is the sample before antibiotic exposures. Orange heatmaps include patients from the ciprofloxacin cohort, blue heatmaps patients from the cotrimoxazole cohort. The bottom row of the heatmaps presents the mean value of each column, here the sample collection time point. The intensity of the color bar reflects the LCRA values, with a deeper color for higher values.
