# Supplementary material for: Distinct impact of antibiotics on the gut microbiome and resistome: a longitudinal multicenter cohort study
Source: BMC Biol. 2019 Sep 18;17:76. doi: 10.1186/s12915-019-0692-y (PMC6749691; doi:10.1186/s12915-019-0692-y)
Supplement: Supplementary file 12 — Figure S8. Trajectories of length-corrected relative abundances of diverse antimicrobial resistance gene classes of both cohorts over the entire observation period. Trajectories of antimicrobial resistance genes quantification by LCRA before treatment (T0), at T1, at T2, and at the end of the observation period (T3) are shown for both antibiotic treatments. Blue data points are measurements at T0, yellow data points at T1, green data points at T2, and dark orange data points at T3. Boxplots indicate the distribution of data. The connecting magenta line shows the means at each time point and their development under treatment. The following ARG classes are depicted: aminoglycosides (AGly), beta-lactamases (Bla), fluoroquinolones (Flq), glycopeptides (Gly), macrolide-lincosamide-streptogramin (MLS), nitroimidazoles (Ntmdz), phenicols (Phe), sulfonamides (Sul), tetracyclines (tet), and trimethoprim (Tmt). CTX-M are cefotaximase enzymes that mediate an ESBL phenotype. (PDF 3083 kb) [file 12915_2019_692_MOESM12_ESM.pdf]

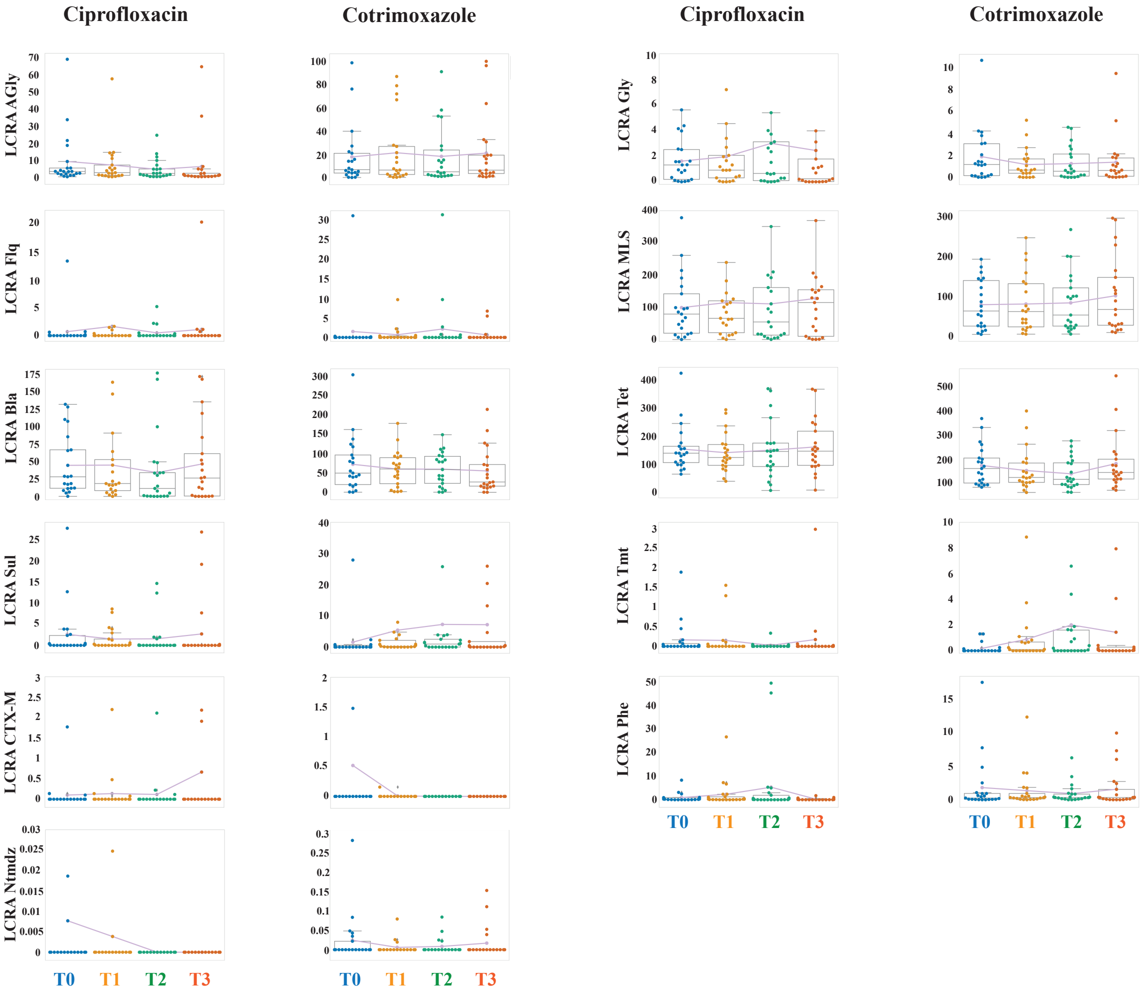

Ciprofloxacin
Cotrimoxazole
Ciprofloxacin
Cotrimoxazole
LCRA AGly
LCRA Flq
LCRA Bla
LCRA Sul
LCRA CTX-M
LCRA Ntmdz
LCRA Gly
LCRA MLS
LCRA Tet
LCRA Tmt
LCRA Phe
T0
T1
T2
T3

Figure S8. Trajectories of length-corrected relative abundances of diverse antimicrobial resistance gene classes of both cohorts over the entire observation period.

Trajectories of antimicrobial resistance genes quantification by LCRA before treatment (T0), at T1, at T2, and at the end of the observation period (T3) are shown for both antibiotic treatments. Blue data points are measurements at T0, yellow data points at T1, green data points at T2, and dark orange data points at T3. Boxplots indicate the distribution of data. The connecting magenta line shows the means at each time point and their development under treatment. The following ARG classes are depicted: aminoglycosides (AGly), beta-lactamases (Bla), fluoroquinolones (Flq), glycopeptides (Gly), macrolide-lincosamide-streptogramin (MLS), nitroimidazoles (Ntmdz), phenicols (Phe), sulfonamides (Sul), tetracyclines (tet), and trimethoprim (Tmt). CTX-M are cefotaximase enzymes that mediate an ESBL phenotype.
