# Supplementary material for: Distinct impact of antibiotics on the gut microbiome and resistome: a longitudinal multicenter cohort study
Source: BMC Biol. 2019 Sep 18;17:76. doi: 10.1186/s12915-019-0692-y (PMC6749691; doi:10.1186/s12915-019-0692-y)
Supplement: Supplementary file 13 — Table S5. Multivariate models of selection pressure estimates on antibiotic resistance genes. (PDF 59 kb) [file 12915_2019_692_MOESM13_ESM.pdf]

Table S5. Multivariate models of selection pressure estimates on antibiotic resistance genes

ARG class	Drug model	Model components	Coefficient (95% CI)	p-value	LR p-value
AGly	Ciprofloxacin	CiproDDD	-0.4 (-1 - 0.2)	0.19	<0.0001
	Cotrimozaxole	CotrimDDD	1.45 (-2.35 - 5.25)	0.45	
		VirosDDD	6.41 (4.37 - 8.45)	<0.001	
Bla	Ciprofloxacin	CiproDDD	0.73 (-1.75 - 3.22)	0.56	0.04
		Leucaemia	60.55 (25.4 - 95.71)	0.001	
	Cotrimozaxole	CotrimDDD	-7.44 (-20.48 - 5.59)	0.25	
		VirosDDD	6.33 (1.04 - 11.61)	0.02	
CTX-M	Ciprofloxacin	CiproDDD	0.05 (-0.01 - 0.12)	0.13	<0.0001
	Cotrimozaxol	CotrimDDD	-0.02 (-0.06 - 0.02)	0.25	
Flq	Ciprofloxacin	CiproDDD	-0.001 (-0.35 - 0.35)	0.99	0.79
	Cotrimozaxole	CotrimDDD	0.15 (-0.74 - 1-05)	0.74	
		Creatinin	2.43 (2.26 - 2.6)	<0.001	
Gly	Ciprofloxacin	CiproDDD	0.04 (-0.21 - 0.29)	0.79	< 0.0001
		Viros	3.27 (0.06 - 6.48)	0.05	
	Cotrimozaxole	CotrimDDD	-0.11 (-0.67 - 0.44)	0.68	
MLS	Ciprofloxacin	CiproDDD	4.22 (-5.91 - 14.35)	0.39	<0.0001
		Viros	-77.07 (-101.54 - -52.58)	<0.001	
	Cotrimozaxole	CotrimDDD	7.76 (-6.9 - 22.52)	0.3	
		Lymphoma	59.98 (27.59 - 92.38)	<0.001	
Ntmdz	Ciprofloxacin	CiproDDD	-0.0008 (-0.002 - 0.00004)	0.06	<0.0001
	Cotrimozaxol	CotrimDDD	-0.006 (-0.018 - 0.017)	0.94	
		Creatinin	-0.003 (-0.005 - -0.0007)	0.01	
		VirosDDD	-0.009 (-0.017 - -0.001)	0.03	
Phe	Ciprofloxacin	CiproDDD	-0.06 (-0.26 - 0.14)	0.55	<0.0001
	Cotrimozaxole	CotrimDDD	0.56 (-0.56 - 1.68)	0.33	
		Creatinin	0.4 (0.27 - 0.53)	<0.001	
Sul	Ciprofloxacin	CiproDDD	-0.13 (-0.42 - 0.17)	0.4	<0.0001
		Bilirubin	7.12 (1.75 - 12.48)	0.009	
	Cotrimozaxole	CotrimDDD	2.34 (0.45 - 4.23)	0.015	
		VirosDDD	29.36 (28.92 - 29.81)	<0.001	
		AFDDD	-0.34 (-0.62 - -0.05)	0.02	
Tet	Ciprofloxacin	CiproDDD	4.57 (-0.83 - 9.97)	0.1	0.94
		Viros	-59.58 (-106.15 - -13.02)	0.01	
	Cotrimozaxole	CotrimDDD	-2.52 (-28.33 - 23.29)	0.85	
		Creatinin	-13.61 (-22.06 - -5.16)	0.002	
Tmt	Ciprofloxacin	CiproDDD	-0.004 (-0.039 - 0.03)	0.78	<0.0001
	Cotrimozaxole	CotrimDDD	0.86 (-0.17 - 1.89)	0.1	
		PPI	0.89 (0.08 - 1.71)	0.03	
		AFDDD	-0.17 (-0.32 - -0.02)	0.03	

ARG, antibiotic resistance gene; 95% CI, 95% confidence interval; LR, likelihood ratio test for coefficient differences; AGly, aminoglycoside ARGs; Bla, beta-lactamases; CTX-M, plasmid-mediated cefotaximases; Flq, fluoroquinolone ARGs; Gly, glycopeptides ARGs; MLS, macrolide-lincosamide-streptogramin ARGs; Ntmdz, nitroimidazole ARG *nimB*; Phe, phenicol ARGs; Sul, Sulfonamide ARGs; Tet, Tetracyclines ARGs; Tmt, Trimethoprim ARGs; CiproDDD, cumulative dose of ciprofloxacin in defined daily doses (DDD); CotrimDDD, cumulative dose of cotrimoxazol in defined daily doses (DDD); VirosDDD, cumulative dose of antiviral agents in defined daily doses (DDD); Leucaemia, leucaemia as underlying disease; Viros, at least one administration of antiviral agents during the observation period; Lymphoma, lymphoma as underlying disease; AFDDD, cumulative dose of antifungals in defined daily doses (DDD); PPI, at least one administration of proton-pump inhibitors during the observation period.

The coefficients denote the increase (positive coefficient) or decrease (negative coefficient) of the respective antibiotic resistance gene class in length corrected relative abundance (LCRA) per unit of the model component. For instance, a coefficient of 1.45 for CotrimDDD within the ARG class AGly (aminoglycosides) means an increase of 1.45 length-corrected relative abundance units of AGly-ARGs per cumulative DDD increase of cotrimoxazole. The p-value denotes the statistical significance of the regression coefficient in a multivariate model, thus corrected for relevant cofactors. Contributing factors are displayed when statistical significant in the multivariate model ($p \leq 0.05$). The LR p-value indicates differences between the antibiotics' coefficients. A Bonferroni corrected LR p-value < 0.002 was regarded a significant difference in the impact of both antibiotics on a specific ARG class. The multivariate coefficient can be identical with the univariate coefficient for antibiotics when no confounding was noted.
