# Supplementary material for: Distinct impact of antibiotics on the gut microbiome and resistome: a longitudinal multicenter cohort study
Source: BMC Biol. 2019 Sep 18;17:76. doi: 10.1186/s12915-019-0692-y (PMC6749691; doi:10.1186/s12915-019-0692-y)
Supplement: Supplementary file 14 — Table S6. Univariate models of selection pressure estimates on antibiotic resistance genes. (PDF 57 kb) [file 12915_2019_692_MOESM14_ESM.pdf]

Table S6. Univariate models of selection pressure estimates on antibiotic resistance genes

ARG class	Drug model	Model components	Coefficient (95% CI)	p-value
AGly	Ciprofloxacin	CiproDDD	-0.4 (-1 - 0.2)	0.19
	Cotrimozaxole	CotrimDDD	1.64 (-2.21 - 5.48)	0.4
		VirosDDD	6.41 (4.37 - 8.45)	<0.001
Bla	Ciprofloxacin	CiproDDD	0.8 (-1.68 - 3.29)	0.53
		Leucaemia	52.66 (16.44 - 88.89)	0.004
		Lymphoma	-40.58 (-74.53 - -6.62)	0.019
	Cotrimozaxole	CotrimDDD	-7.24 (-20.05 - 5.5)	0.25
		VirosDDD	6.33 (1.04 - 11.61)	0.021
CTX-M	Ciprofloxacin	CiproDDD	0.05 (-0.01 - 0.12)	0.13
	Cotrimozaxol	CotrimDDD	-0.02 (-0.06 - 0.02)	0.25
Flq	Ciprofloxacin	CiproDDD	-0.001 (-0.35 - 0.35)	0.99
	Cotrimozaxole	CotrimDDD	-0.04 (-0.84 - 0.77)	0.93
		Creatinin	2.43 (2.26 - 2.6)	<0.001
Gly	Ciprofloxacin	CiproDDD	0.04 (-0.22 - 0.29)	0.79
		Viros	3.27 (0.06 - 6.48)	0.045
	Cotrimozaxole	CotrimDDD	-0.11 (-0.67 - 0.44)	0.68
MLS	Ciprofloxacin	CiproDDD	4 (-6.03 - 14.03)	0.42
		Viros	-77.07 (-101.54 - -52.58)	<0.001
	Cotrimozaxole	CotrimDDD	7.95 (-6.48 - 22.38)	0.28
		Lymphoma	60.74 (29.38 - 92.1)	<0.001
		VirosDDD	6.94 (0.18 - 13.7)	0.04
Ntmdz	Ciprofloxacin	CiproDDD	-0.0008 (-0.002 - 0.00004)	0.06
	Cotrimozaxol	CotrimDDD	-0.002 (-0.02 - 0.01)	0.75
		Creatinin	-0.003 (-0.005 - -0.0007)	0.01
		VirosDDD	-0.005 (-0.009 - -0.001)	0.006
Phe	Ciprofloxacin	CiproDDD	-0.06 (-0.26 - 0.14)	0.55
	Cotrimozaxole	CotrimDDD	-0.06 (-0.89 - 0.77)	0.89
		Creatinin	0.44 (0.27 - 0.62)	<0.001
		Bilirubin	-2.26 (-4.33 - -0.21)	0.03
		VirosDDD	0.46 (0.25 - 0.67)	<0.001
Sul	Ciprofloxacin	CiproDDD	0.14 (-0.29 - 0.56)	0.52
		Bilirubin	7.12 (1.75 - 12.48)	0.009
	Cotrimozaxole	CotrimDDD	2.89 (0.12 - 5.67)	0.041
		VirosDDD	29.42 (29.02 - 29.82)	<0.001
		AFDDD	-0.68 (-1.35 - -0.02)	0.035
Tet	Ciprofloxacin	CiproDDD	2.34 (-2.57 - 7.26)	0.35
		Viros	-66.68 (-113.32 - -20.05)	0.005
		AFDDD	-5.94 (-11.1 - -0.78)	0.02
	Cotrimozaxole	CotrimDDD	0.92 (-18.81 - 20.66)	0.93
		Creatinin	-13.39 (-21.33 - -5.44)	0.001
		VirosDDD	21.35 (12.79 - 29.92)	<0.001
Tmt	Ciprofloxacin	CiproDDD	-0.004 (-0.039 - 0.03)	0.78
	Cotrimozaxole	CotrimDDD	0.88 (-0.05 - 1.8)	0.064
		PPI	0.88 (0.08 - 1.67)	0.03
		AFDDD	-0.17 (-0.32 - -0.02)	0.03

ARG, antibiotic resistance gene; 95% CI, 95% confidence interval; LR, likelihood ratio test for coefficient differences; AGly, aminoglycoside ARGs; Bla, beta-lactamases; CTX-M, plasmid-mediated cefotaximases; Flq, fluoroquinolone ARGs; Gly, glycopeptides ARGs; MLS, macrolide-lincosamide-streptogramin ARGs; Ntmdz, nitroimidazole ARG *nimB*; Phe, phenicol ARGs; Sul, Sulfonamide ARGs; Tet, Tetracyclines ARGs; Tmt, Trimethoprim ARGs; CiproDDD, cumulative dose of ciprofloxacin in defined daily doses (DDD); CotrimDDD, cumulative dose of cotrimoxazol in defined daily doses (DDD); VirosDDD, cumulative dose of antiviral agents in defined daily doses (DDD); Leucaemia, leucaemia as underlying disease; Viros, at least one administration of antiviral agents during the observation period; Lymphoma, lymphoma as underlying disease; AFDDD, cumulative dose of antifungals in defined daily doses (DDD); PPI, at least one administration of proton-pump inhibitors during the observation period.

The coefficients denote the increase (positive coefficient) or decrease (negative coefficient) of the respective antibiotic resistance gene class in length corrected relative abundance (LCRA) per unit of the model component. For instance, a coefficient of 1.64 for CotrimDDD within the ARG class AGly (aminoglycosides) means an increase of 1.64 length-corrected relative abundance units of AGly-ARGs per cumulative DDD increase of cotrimoxazole. The p-value denotes the statistical significance of the regression coefficient in a univariate model.
