# Supplementary material for: Distinct impact of antibiotics on the gut microbiome and resistome: a longitudinal multicenter cohort study
Source: BMC Biol. 2019 Sep 18;17:76. doi: 10.1186/s12915-019-0692-y (PMC6749691; doi:10.1186/s12915-019-0692-y)
Supplement: Supplementary file 15 — Table S7. Baseline disparities in the intestinal resistome between BEC groups. (PDF 53 kb) [file 12915_2019_692_MOESM15_ESM.pdf]

Table S7. Baseline disparities in the intestinal resistome between BEC groups

ARG class	Drug	Mean (Median), n Decrease Group	Mean (Median), n Increase Group	p-value
AGly	Ciprofloxacin	12.3 (3.47), 14	1.26 (1.36), 6	0.01
	Cotrimoxazole	18.44 (11), 12	16.84 (4.38), 9	0.89
Bla	Ciprofloxacin	40.03 (23.35), 10	49.37 (38.98), 10	0.65
	Cotrimoxazole	90.19 (49.18), 11	51.11 (37.11), 10	0.21
CTX-M	Ciprofloxacin	0.008 (0), 17	0.64 (0.14), 3	0.008
	Cotrimoxazole	0.05 (0), 21	n.a., 0	n.a.
Flq	Ciprofloxacin	0.78 (0), 17	0.26 (0.17), 3	0.03*
	Cotrimoxazole	1.72 (0), 18	0.1 (0), 3	0.71
Gly	Ciprofloxacin	1.47 (1.12), 14	1.94 (1.45), 6	0.59
	Cotrimoxazole	2.63 (1.39), 13	0.64 (0.25), 8	0.04
MLS	Ciprofloxacin	123.86 (95.07), 10	75.46 (74.09), 10	0.71
	Cotrimoxazole	81.04 (75.86), 8	72.36 (59.85), 13	0.76
Ntmdz	Ciprofloxacin	0.008 (0), 20	n.a., 0	n.a.
	Cotrimoxazole	0.024 (0), 19	0.024 (0.024), 2	0.99
Phe	Ciprofloxacin	1.23 (0.26), 14	0.18 (0.11), 6	0.28
	Cotrimoxazole	2.66 (0.77), 14	0.07 (0.06), 7	0.007
Sul	Ciprofloxacin	2.52 (0), 19	3.83 (3.83), 1	0.85
	Cotrimoxazole	0.36 (0), 11	2.94 (0), 10	0.87
Tet	Ciprofloxacin	162.95 (135.31), 9	138.49 (129.75), 11	0.53
	Cotrimoxazole	190.16 (178.17), 10	158.25 (138.17), 11	0.38
Tmt	Ciprofloxacin	0.18 (0), 18	0 (0), 2	0.59
	Cotrimoxazole	0.29 (0), 13	0.004 (0), 8	0.15

ARG, antibiotic resistance gene; AGly, aminoglycoside ARGs; Bla, beta-lactamases; CTX-M, plasmid-mediated cefotaximases; Flq, fluoroquinolone ARGs; Gly, glycopeptide ARGs; MLS, macrolide-lincosamide-streptogramin ARGs; Ntmdz, nitroimidazole ARG *nimB*; Phe, phenicol ARGs; Sul, Sulfonamide ARGs; Tet, Tetracyclines ARGs; Tmt, Trimethoprim ARGs.

We have performed a comparison between length-corrected relative abundance (LCRA) from the last time point and the baseline (T3 - T0, BEC) for each patient. If a parameter did not change its BEC value or decreased (BEC value ≤ 0), the patient was part of the “Decrease Group”. If the BEC value was > 0, the patient was classified to belong to the “Increase Group”. Mean and median of baseline LCRA for each class of antimicrobial resistance genes from both BEC groups are documented and hypothesis testing regarding a statistically significant difference was performed (p-value). ”n” denotes the respective group size.

*The fluoroquinolone ARG group is skewed in their distribution and has unequal variances, leading to conflicting values for means and medians. This is likely due to the relatively low numbers of non-zero values. Despite the statistical significance in the Wilcoxon ranksum test, we have refrained from any conclusion. This is also due to the fact, that the medians seem not to be different from each other (non-parametric *k*-sample test on equality of medians, p = 0.159 after continuity correction).
