# Supplementary material for: Distinct impact of antibiotics on the gut microbiome and resistome: a longitudinal multicenter cohort study
Source: BMC Biol. 2019 Sep 18;17:76. doi: 10.1186/s12915-019-0692-y (PMC6749691; doi:10.1186/s12915-019-0692-y)
Supplement: Supplementary file 16 — Figure S9. Correlation matrix between gut resistome fractions and taxonomic units. A Kendall’s rank correlation matrix between taxonomic units (phylum level) and ARG classes for the ciprofloxacin cohort (A) und the cotrimoxazole cohort (B). The following ARG classes are depicted: aminoglycosides (AGly), beta-lactamases (Bla), fluoroquinolones (Flq), glycopeptides (Gly), macrolide-lincosamide-streptogramin (MLS), nitroimidazoles (Ntmdz), phenicols (Phe), sulfonamides (Sul), tetracyclines (tet), and trimethoprim (Tmt). (PDF 1180 kb) [file 12915_2019_692_MOESM16_ESM.pdf]

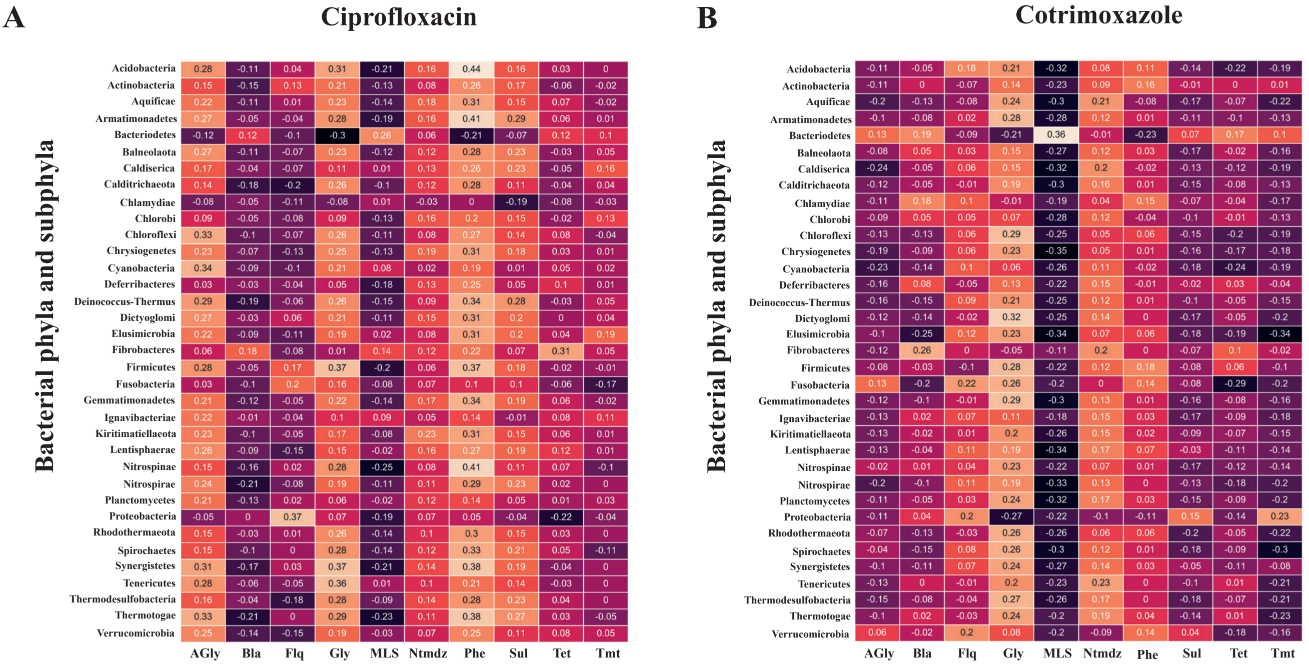
A
Ciprofloxacin
B
Cotrimoxazole
Bacterial phyla and subphyla
Acidobacteria
Actinobacteria
Aquificae
Armatimonadetes
Bacteriodetes
Balneolaota
Caldiserica
Calditrichaeota
Chlamydiae
Chlorobi
Chloroflexi
Chrysiogenetes
Cyanobacteria
Deferribacteres
Deinococcus-Thermus
Dictyoglomi
Elusimicrobia
Fibrobacteres
Firmicutes
Fusobacteria
Gemmatimonadetes
Ignavibacteriae
Kiritimatiellaeota
Lentisphaerae
Nitrospinae
Nitrospirae
Planctomycetes
Proteobacteria
Rhodothermaeota
Spirochaetes
Synergistetes
Tenericutes
Thermodesulfobacteria
Thermotogae
Verrucomicrobia
AGly
Bla
Flq
Gly
MLS
Ntmdz
Phe
Sul
Tet
Tmt

Figure S9. Correlation matrix between gut resistome fractions and taxonomic units

A Kendall's rank correlation matrix between taxonomic units (phylum level) and ARG classes for the ciprofloxacin cohort (A) und the cotrimoxazole cohort (B). The following ARG classes are depicted: aminoglycosides (AGly), beta-lactamases (Bla), fluoroquinolones (Flq), glycopeptides (Gly), macrolide-lincosamide-streptogramin (MLS), nitroimidazoles (Ntmdz), oxazolidinones (Oxzln), phenicols (Phe), sulfonamides (Sul), tetracyclines (tet), and trimethoprim (Tmt).
