# Supplementary material for: Distinct impact of antibiotics on the gut microbiome and resistome: a longitudinal multicenter cohort study
Source: BMC Biol. 2019 Sep 18;17:76. doi: 10.1186/s12915-019-0692-y (PMC6749691; doi:10.1186/s12915-019-0692-y)
Supplement: Supplementary file 17 — Table S8. Kendall’s rank correlation between gut microbiome species and gut resistome. (PDF 53 kb) [file 12915_2019_692_MOESM17_ESM.pdf]

Table S8. Kendall's rank correlation between gut microbiome species and gut resistome

Taxon	ARG class	tau correlation coefficient	p-value
Cotrimoxazole cohort			
Klebsiella variicola	Tmt	0.29	0.0003
Klebsiella variicola	Sul	0.27	0.0006
Klebsiella aerogenes	Tmt	0.28	0.0004
Klebsiella aerogenes	Sul	0.24	0.0022
Klebsiella oxytoca	Sul	0.18	0.02
Citrobacter youngae	Sul	0.31	0.0001
Citrobacter youngae	Tmt	0.28	0.0006
Citrobacter pasteurii	Tmt	0.3	0.0007
Citrobacter europaeus	Sul	0.25	0.0038
Citrobacter farmerii	Tmt	0.24	0.005
Citrobacter farmerii	Sul	0.21	0.01
Citrobacter freundii	Tmt	0.2	0.012
Citrobacter werkmanii	Tmt	0.2	0.016
Serratia marcescens	Sul	0.19	0.017
Enterobacter asburiae	Sul	0.31	0.0001
Enterobacter asburiae	Tmt	0.26	0.0016
Enterobacter cloacae	Tmt	0.26	0.0012
Enterobacter cloacae	Sul	0.24	0.01
Ciprofloxacin cohort			
Escherichia coli	Flq	0.22	0.013
Citrobacter amalonaticus	Flq	0.35	0.0001
Citrobacter youngae	Flq	0.32	0.0003
Citrobacter farmerii	Flq	0.33	0.0006
Enterobacter cloacae	Flq	0.35	0.0007
Serratia marcescens	Flq	0.36	0.00004
Staphylococcus aureus	Flq	0.36	0.00005
Staphylococcus saccharolyticus	Flq	0.29	0.0017

ARG, antibiotic resistance gene; Flq, fluoroquinolone ARGs; Sulfonamide ARGs; Tmt, Trimethoprim ARGs.

A positive coefficient indicates a parallel increase or decrease of taxa and resistance genes. For instance, *Klebsiella varicola* relative abundance increases with the length corrected relative abundance of trimethoprim resistances genes (tau = 0.29, p = 0.003). Since we observed a strong increase of trimethoprim resistances genes under cotrimoxazole treatment, a parallel increase of *Klebsiella varicola* can be suggested. Another interesting example is *Staphylococcus saccharolyticus* which is positively correlated with fluoroquinolone ARGs. We observed a high number of novel detections of this species under ciprofloxacin treatment (Figure 1E), suggesting a link between the antibiotic treatment and relative species abundance.
