# Supplementary material for: Distinct impact of antibiotics on the gut microbiome and resistome: a longitudinal multicenter cohort study
Source: BMC Biol. 2019 Sep 18;17:76. doi: 10.1186/s12915-019-0692-y (PMC6749691; doi:10.1186/s12915-019-0692-y)
Supplement: Supplementary file 18 — Table S9. Baseline disparities in the intestinal plasmidome between BEC groups. (PDF 47 kb) [file 12915_2019_692_MOESM18_ESM.pdf]

Table S9. Baseline disparities in the intestinal plasmidome between BEC groups

Plasmidome parameter	Drug	Mean (Median), n Decrease Group	Mean (Median), n Increase Group	p-value
Plasmid diversity	Ciprofloxacin	6.28 (6.6), 19	4.31 (4.31), 1	0.19
	Cotrimoxazole	6.66 (6.48), 13	6.29 (6.78), 8	0.83
Plasmid evenness	Ciprofloxacin	0.029 (0.027), 11	0.015 (0.015), 9	0.004
	Cotrimoxazole	0.026 (0.026), 14	0.016 (0.013), 7	0.05
Plasmid abundance	Ciprofloxacin	912.41 (846.86), 17	668.18 (747.96), 3	0.18
	Cotrimoxazole	1038.4 (1039.98), 13	1113.27 (1147.27), 8	0.66
Plasmid abundance (Proteobacteria)	Ciprofloxacin	186.34 (165.34), 17	93.66 (111.86), 3	0.17
	Cotrimoxazole	200.66 (186.23), 10	187.61 (192.56), 11	0.73

We have performed a comparison between data from the last time point and the baseline (T3 - T0, BEC). If a parameter did not change its BEC value or decreased (BEC value ≤ 0), the patient was part of the “Decrease Group”. If the BEC value was > 0, the patient was classified to belong to the “Increase Group”. Mean and median of baseline parameters from both groups are documented and hypothesis testing regarding a statistically significant difference was performed (p-value). ”n” denotes the respective group size. Plasmid abundance is expressed as normalized plasmid coverage.
