# Supplementary material for: Distinct impact of antibiotics on the gut microbiome and resistome: a longitudinal multicenter cohort study
Source: BMC Biol. 2019 Sep 18;17:76. doi: 10.1186/s12915-019-0692-y (PMC6749691; doi:10.1186/s12915-019-0692-y)
Supplement: Supplementary file 19 — Figure S10. Trajectories of plasmid abundance, plasmid diversity and plasmid evenness of both cohorts over the entire observation period. Trajectories of plasmid abundance, plasmid diversity and plasmid evenness before treatment (T0), at T1, at T2, and at the end of the observation period (T3) are shown for both antibiotic treatments. Blue data points are measurements at T0, yellow data points at T1, green data points at T2, and dark orange data points at T3. Boxplots indicate the distribution of data. The connecting magenta line shows the means at each time point and their development under treatment. (PDF 1651 kb) [file 12915_2019_692_MOESM19_ESM.pdf]

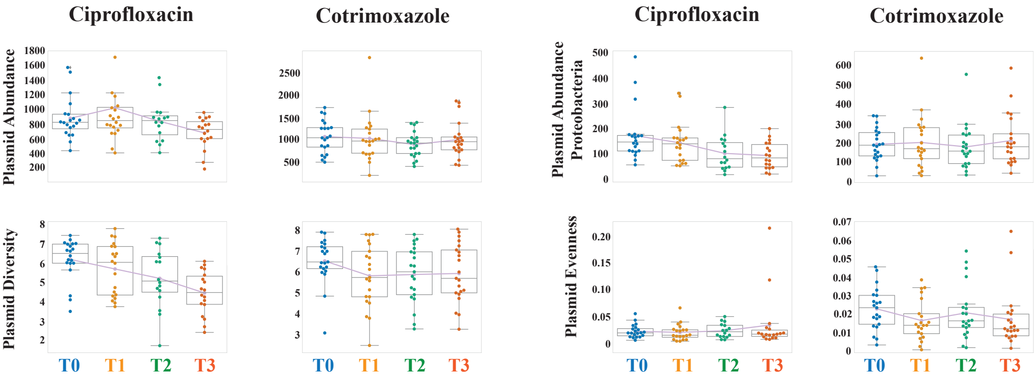

Ciprofloxacin
Cotrimoxazole
Ciprofloxacin
Cotrimoxazole
Plasmid Abundance
Plasmid Abundance Proteobacteria
Plasmid Diversity
Plasmid Evenness
T0
T1
T2
T3

Figure S10. Trajectories of plasmid abundance, plasmid diversity and plasmid evenness of both cohorts over the entire observation period.

Trajectories of plasmid abundance, plasmid diversity and plasmid evenness before treatment (T0), at T1, at T2, and at the end of the observation period (T3) are shown for both antibiotic treatments. Blue data points are measurements at T0, yellow data points at T1, green data points at T2, and dark orange data points at T3. Boxplots indicate the distribution of data. The connecting magenta line shows the means at each time point and their development under treatment.
