# Supplementary material for: Distinct impact of antibiotics on the gut microbiome and resistome: a longitudinal multicenter cohort study
Source: BMC Biol. 2019 Sep 18;17:76. doi: 10.1186/s12915-019-0692-y (PMC6749691; doi:10.1186/s12915-019-0692-y)
Supplement: Supplementary file 20 — Table S10. Multivariate models of selection pressure estimates on the intestinal plasmidome. (PDF 46 kb) [file 12915_2019_692_MOESM20_ESM.pdf]

Table S10. Multivariate models of selection pressure estimates on the intestinal plasmidome

Plasmidome variable	Drug model	Model components	Coefficient (95% CI)	p-value	LR p-value
Plasmid Shannon diversity	Ciprofloxacin	CiproDDD	-0.23 (-0.29 - -0.17)	<0.001	1
		Creatinin	0.93 (0.15 - 1.72)	0.02	
	Cotrimoxazole	CotrimDDD	-0.25 (-0.53 - 0.03)	0.08	
		Lymphoma	-1.13 (-1.45 - -0.81)	<0.001	
		VirosDDD	-0.51 (-0.64 - -0.37)	<0.001	
Plasmid evenness	Ciprofloxacin	CiproDDD	0.001 (-0.002 - 0.005)	0.46	<0.0001
	Cotrimoxazole	CotrimDDD	-0.003 (-0.007 - 0.001)	0.19	
		Lymphoma	-0.01 (-0.02 - -0.004)	0.001	
		Viros	-0.005 (-0.009 - -0.001)	0.014	
Plasmid abundance	Ciprofloxacin	CiproDDD	-36.18 (-57.14 - -15.21)	0.002	0.02
		Viros	58.53 (11.36 - 105.7)	0.02	
	Cotrimoxazole	CotrimDDD	-45.65 (-123.64 - 32.33)	0.24	
		VirosDDD	-67.06 (-98.7 - -35.43)	<0.001	
Plasmid abundance (Proteobacteria)	Ciprofloxacin	CiproDDD	-9.9 (-16.17 - -3.63)	0.002	0.02
	Cotrimoxazole	CotrimDDD	2.12 (-21.95 - 26.21)	0.86	
		AF	31.64 (2.65 - 60.63)	0.03	
		VirosDDD	-24.55 (-34.31 - -14.78)	<0.001	

95% CI, 95% confidence interval; LR, likelihood ratio test for coefficient differences; CiproDDD, cumulative dose of ciprofloxacin in defined daily doses (DDD); CotrimDDD, cumulative dose of cotrimoxazole in defined daily doses (DDD); VirosDDD, cumulative dose of antiviral agents in defined daily doses (DDD); Lymphoma, lymphoma as underlying disease; AF, at least one administration of antifungals during the observation period; Viros, at least one administration of antiviral agents during the observation period; Platelets, platelet count.

The coefficients denote the increase (positive coefficient) or decrease (negative coefficient) of the plasmid diversity/evenness/abundance per unit of the model component. For instance, a coefficient of -0.23 for CiproDDD regarding plasmid diversity means a decrease of 0.23 units Shannon diversity per cumulative DDD increase of ciprofloxacin. The p-value denotes the statistical significance of the regression coefficient in a multivariate model, thus corrected for relevant cofactors. Contributing factors are displayed when statistical significant in the multivariate model ($p \leq 0.05$). The LR p-value indicates differences between the antibiotics' coefficients. A Bonferroni corrected LR p-value < 0.002 was regarded a significant difference in the impact of both antibiotics on a specific plasmidome variable. Plasmid abundance is expressed as normalized plasmid coverage. The multivariate coefficient can be identical with the univariate coefficient for antibiotics when no confounding was noted.
