# Supplementary material for: Distinct impact of antibiotics on the gut microbiome and resistome: a longitudinal multicenter cohort study
Source: BMC Biol. 2019 Sep 18;17:76. doi: 10.1186/s12915-019-0692-y (PMC6749691; doi:10.1186/s12915-019-0692-y)
Supplement: Supplementary file 21 — Table S11. Univariate models of selection pressure estimates on the intestinal plasmidome. (PDF 43 kb) [file 12915_2019_692_MOESM21_ESM.pdf]

Table S11. Univariate models of selection pressure estimates on the intestinal plasmidome

Plasmidome variable	Drug model	Model components	Coefficient (95% CI)	p-value
Plasmid Shannon diversity	Ciprofloxacin	CiproDDD	-0.21 (-0.27 - -0.14)	<0.001
		Creatinin	0.93 (0.15 - 1.71)	0.02
	Cotrimoxazole	CotrimDDD	-0.27 (-0.54 - 0.02)	0.07
		Leucaemia	1.14 (0.31 - 1.97)	0.007
		Lymphoma	-1.5 (-2.08 - -0.92)	<0.001
		VirosDDD	-0.54 (-0.67 - -0.41)	<0.001
Plasmid evenness	Ciprofloxacin	CiproDDD	0.001 (-0.002 - 0.005)	0.46
	Cotrimoxazole	CotrimDDD	-0.003 (-0.007 - 0.001)	0.19
		Lymphoma	-0.01 (-0.02 - -0.005)	0.001
		Viros	-0.006 (-0.01 - -0.002)	0.002
Plasmid abundance	Ciprofloxacin	CiproDDD	-36.02 (-56.68 - -15.37)	0.002
		Viros	58.53 (11.36 - 105.7)	0.02
	Cotrimoxazole	CotrimDDD	-47.81 (-124.33 - 28.71)	0.21
		VirosDDD	-67.07 (-98.7 - -35.43)	<0.001
Plasmid abundance (Proteobacteria)	Ciprofloxacin	CiproDDD	-9.86 (-16.2 - -3.53)	0.002
		Lymphoma	-58.39 (-97.81 - -18.97)	0.004
		Platelets	-0.0002 (-0.0003 - -0.00008)	0.002
		PPI	-47.46 (-84.46 - -10.45)	0.01
	Cotrimoxazole	CotrimDDD	3.89 (-17.59 - 25.38)	0.71
		AF	32.67 (4.18 - 61.16)	0.03
		VirosDDD	-25.6 (-34.47 - -16.74)	<0.001

95% CI, 95% confidence interval; LR, likelihood ratio test for coefficient differences; CiproDDD, cumulative dose of ciprofloxacin in defined daily doses (DDD); CotrimDDD, cumulative dose of cotrimoxazole in defined daily doses (DDD); VirosDDD, cumulative dose of antiviral agents in defined daily doses (DDD); Lymphoma, lymphoma as underlying disease; Leucaemia, leucaemia as underlying disease; AF, at least one administration of antifungals during the observation period; Viros, at least one administration of antiviral agents during the observation period; Platelets, platelet count; PPI, at least one administration of proton-pump inhibitors during the observation period.

The coefficients denote the increase (positive coefficient) or decrease (negative coefficient) of the plasmid diversity/evenness/abundance per unit of the model component. For instance, a coefficient of -0.21 for CiproDDD regarding plasmid diversity means a decrease of 0.21 units Shannon diversity per cumulative DDD increase of ciprofloxacin. The p-value denotes the statistical significance of the regression coefficient in a univariate model. Plasmid abundance is expressed as normalized plasmid coverage.
