# Supplementary material for: Distinct impact of antibiotics on the gut microbiome and resistome: a longitudinal multicenter cohort study
Source: BMC Biol. 2019 Sep 18;17:76. doi: 10.1186/s12915-019-0692-y (PMC6749691; doi:10.1186/s12915-019-0692-y)
Supplement: Supplementary file 22 — Table S12. Complete list of inclusion and exclusion criteria (PDF 36 kb) [file 12915_2019_692_MOESM22_ESM.pdf]

Table S12. Complete list of inclusion and exclusion criteria

**Inclusion Criteria**
Female and male patients ≥ 18 years
Patients with an underlying hemato-oncological disease with an expected neutropenia ≥ 7 days
Inpatient or outpatient treatment in one of both study centers
Written consent of study participants
**Exclusion criteria**
Antibiotic treatment within the previous 30 days
Need for antibiotic medication other than ciprofloxacin or cotrimoxazole during the observation period
Pregnancy
Patients with chronic viral diseases (HIV, HBV, HCV)
Comorbidities that render a patients understanding of the study's concepts, objectives and potential consequences impossible
A high likelihood that a patient would not comply with the study protocol
