# Supplementary material for: Distinct impact of antibiotics on the gut microbiome and resistome: a longitudinal multicenter cohort study
Source: BMC Biol. 2019 Sep 18;17:76. doi: 10.1186/s12915-019-0692-y (PMC6749691; doi:10.1186/s12915-019-0692-y)
Supplement: Supplementary file 23 — Table S13. List of concomitant drugs in both study cohorts (PDF 46 kb) [file 12915_2019_692_MOESM23_ESM.pdf]

Table S13. List of concomitant drugs in both study cohorts

Ciprofloxacin cohort	Cotrimoxazole cohort
Virostatic agents	
• acyclovir<br>• ribavirin	• valaciclovir
Antifungals	
• posaconazole<br>• amphotericin B<br>• pentamidine<br>• fluconazole	• posaconazole<br>• amphotericin B
Proton pump inhibitors	
• pantoprozole	• pantoprozole
Anti-cancer substances	
• cytarabine<br>• rituximab<br>• cyclophosphamide<br>• etoposide<br>• vincristine<br>• doxorubicin<br>• bortezomib<br>• cisplatin<br>• imatinib<br>• hydroxyurea<br>• idarubicin<br>• lenalidomide<br>• daunorubicin<br>• decitabine<br>• arsenic trioxide	• cytarabine<br>• rituximab<br>• cyclophosphamide<br>• etoposide<br>• vincristine<br>• doxorubicin<br>• bortezomib<br>• procarbazine<br>• mitoxantrone<br>• bleomycin<br>• ifosfamide<br>• elotuzumab<br>• methotrexate
Laxatives	
• lactulose<br>• polyethylene glycol (macrogol)<br>• sodium picosulfate	• lactulose<br>• polyethylene glycol (macrogol)<br>• sodium picosulfate
Cholesterol-lowering substances	
• simvastatin	
Immunosuppressives	
• dexamethasone<br>• prednisolone	• dexamethasone<br>• prednisone
Others	
• ranitidine<br>• retinoic acid	• ranitidine
